# Artificial intelligence in the risk prediction models of cardiovascular disease and development of an independent validation screening tool: a systematic review

**DOI:** 10.1186/s12916-024-03273-7

**Published:** 2024-02-05

**Authors:** Yue Cai, Yu-Qing Cai, Li-Ying Tang, Yi-Han Wang, Mengchun Gong, Tian-Ci Jing, Hui-Jun Li, Jesse Li-Ling, Wei Hu, Zhihua Yin, Da-Xin Gong, Guang-Wei Zhang

**Affiliations:** 1 China Medical University, Shenyang, 110122 China; 2 Digital Health China Co. Ltd, Beijing, 100089 China; 3 Smart Hospital Management Department, the First Hospital of China Medical University, Shenyang, 110001 China; 4 Shenyang Medical & Film Science and Technology Co. Ltd., Shenyang, 110001 China; 5 Enduring Medicine Smart Innovation Research Institute, Shenyang, 110001 China; 6 Institute of Genetic Medicine, School of Life Science, State Key Laboratory of Biotherapy, Sichuan University, Chengdu, 610065 China; 7 Bayi Orthopedic Hospital, Chengdu, 610017 China; 8 Department of Epidemiology, School of Public Health, China Medical University, Shenyang, 110122 China; 9 The Internet Hospital Branch of the Chinese Research Hospital Association, Beijing, 100006 China

**Keywords:** Artificial intelligence, Cardiovascular disease, Machine learning, Risk prediction models

## Abstract

**Background:**

A comprehensive overview of artificial intelligence (AI) for cardiovascular disease (CVD) prediction and a screening tool of AI models (AI-Ms) for independent external validation are lacking. This systematic review aims to identify, describe, and appraise AI-Ms of CVD prediction in the general and special populations and develop a new independent validation score (IVS) for AI-Ms replicability evaluation.

**Methods:**

PubMed, Web of Science, Embase, and IEEE library were searched up to July 2021. Data extraction and analysis were performed for the populations, distribution, predictors, algorithms, etc. The risk of bias was evaluated with the prediction risk of bias assessment tool (PROBAST). Subsequently, we designed IVS for model replicability evaluation with five steps in five items, including transparency of algorithms, performance of models, feasibility of reproduction, risk of reproduction, and clinical implication, respectively. The review is registered in PROSPERO (No. CRD42021271789).

**Results:**

In 20,887 screened references, 79 articles (82.5% in 2017–2021) were included, which contained 114 datasets (67 in Europe and North America, but 0 in Africa). We identified 486 AI-Ms, of which the majority were in development (*n* = 380), but none of them had undergone independent external validation. A total of 66 idiographic algorithms were found; however, 36.4% were used only once and only 39.4% over three times. A large number of different predictors (range 5–52,000, median 21) and large-span sample size (range 80–3,660,000, median 4466) were observed. All models were at high risk of bias according to PROBAST, primarily due to the incorrect use of statistical methods. IVS analysis confirmed only 10 models as “recommended”; however, 281 and 187 were “not recommended” and “warning,” respectively.

**Conclusion:**

AI has led the digital revolution in the field of CVD prediction, but is still in the early stage of development as the defects of research design, report, and evaluation systems. The IVS we developed may contribute to independent external validation and the development of this field.

**Supplementary Information:**

The online version contains supplementary material available at 10.1186/s12916-024-03273-7.

## Background

The surge in cardiovascular diseases (CVDs) has become a global challenge with a steadily climbing trend of cardiovascular deaths from 12.1 million in 1990 to 18.6 million in 2019 [1, 2]. Risk prediction, a primary strategy in addressing this worldwide problem, has brought significant benefits to some developed countries through the improvement of the effectiveness of life intervention and reduction of economic burden [3, 4]. Therefore, risk prediction has been expected as an efficient way to achieve World Health Organization (WHO) goals for reducing CVD-related mortality by 25% by 2025, and some classic CVD prediction models (e.g., the Framingham [5] and SCORE [6], referred to as traditional models [T-Ms] in this study) has been incorporated into clinical guidelines by the European Society of Cardiology (ESC) and the American College of Cardiology/American Heart Association (ACC/AHA) [7, 8].

Artificial intelligence (AI), encompassing machine learning (ML) and deep learning (DL), is a field within computer science dedicated to the development of computational systems capable of performing tasks that traditionally necessitate human intelligence, such as learning, reasoning, problem-solving, perception, language comprehension, and decision-making. The application of AI in the healthcare sector, including disease risk prediction, is rapidly advancing and playing an increasingly significant role [9–13]. Alongside the substantial transformations driven by AI in this domain, it also introduces a spectrum of challenges and issues, including concerns related to ethics, legality, data privacy, security, bias, fairness, transparency, and explainability [14–20]. At this critical juncture in the AI field, characterized by a coexistence of challenges and opportunities in the era of big data, AI-driven disease risk prediction stands ready to harness immense potential and address substantial needs [11, 21]. It has demonstrated notable superiority over the T-Ms, owing to its more robust data-processing capability, fewer condition restrictions, and better performance [11], thereby providing a more promising predictive strategy for CVDs.

However, a comprehensive and systematic overview of AI for CVD prediction is still lacking, despite the field has witnessed several recent comparative reviews that tend to emphasize specific aspects. For instance, Suri et al. provided a comprehensive summary of ML paradigms with a technical emphasis [22]. Azmi et al. focused on emphasis on comparing the predictive performance of various ML-based classification algorithms using medical big data [23]. Infante et al. and Assadi et al. primarily reviewed the contributions of cardiac computed tomography angiography and cardiac magnetic resonance to AI-CVD prediction [24, 25]. Triantafyllidis et al. conducted a review on the impact of DL on the diagnosis, management, and treatment of major chronic diseases, including cardiovascular disease [26]. Zhao et al. only observed social determinants contributing to AI-CVD prediction [27]. Liu et al. compared the ML and traditional approaches for atherosclerotic CVD risk prognostication [28]. These articles provide limited insights for a comprehensive understanding of the current state of this field. Therefore, in reference to previously published reviews that elucidate the development status of T-Ms for CVD prediction [29], we conducted this summarization work and attempted to explore potential solutions to address the current challenges.

## Methods

We conducted this systematic review using the CHARMS checklist. This review has been registered in the international prospective register of systematic reviews (PROSPERO), with the registration number CRD42021271789, where all updates of the review will also be recorded. This review followed the Preferred Reporting Items for Systematic reviews and Meta-Analysis (PRISMA) statement (Additional file 1). Patients and the public were not involved in setting of the research question, designing or implementing the study, or in interpreting or writing of the results.

### Literature search

A literature search was conducted in PubMed, Web of Science, Embase, and IEEE, using search terms to identify primary articles focused on the development and/or validation of AI in predicting incident CVD up to July 2021. A cross-reference check was performed for all reviews on CVD prediction models identified by our search. Search strategies are described in Additional file 2: Text 1.

### Eligibility criteria

**Fig. 1:**
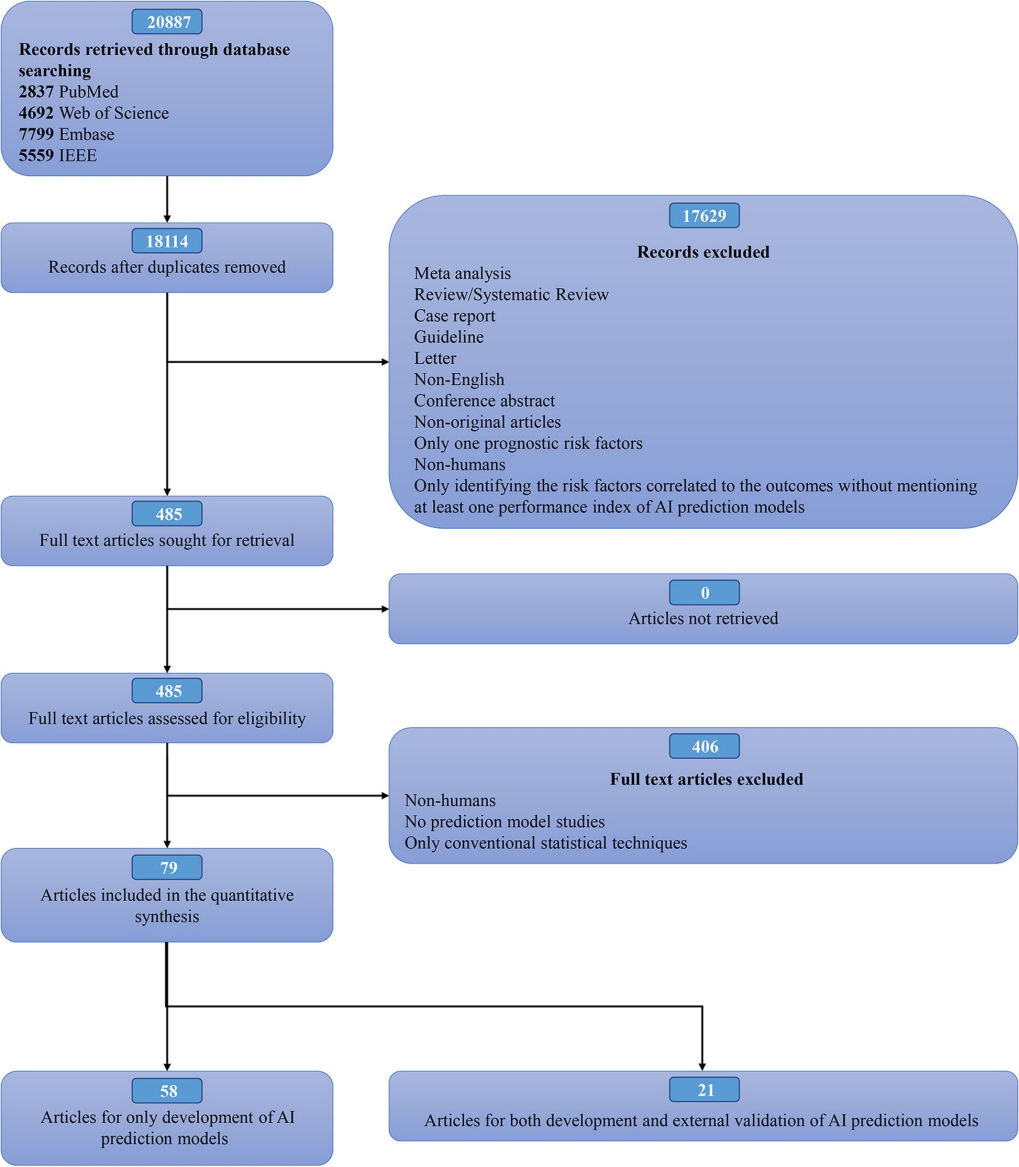
The flow diagram for the literature search performed in the present study

We included only original research on risk prediction models for humans with full text in English, excluding studies that (1) are for clustering and outcome classification and (2) in the postoperative or perioperative period of cardiac surgery or non-cardiac surgery. The detailed process and criteria are shown in Fig. 1.

### Screening process

Two independent reviewers screened the titles and abstracts. The corresponding full texts were retrieved and reviewed after identifying potentially eligible articles. Any disagreements during this process were resolved through discussion among all team members to reach a consensus.

### Data extraction and critical appraisal

The list of extracted items was based on the CHARMS checklist. Two independent reviewers extracted the data, with any discrepancies being resolved through discussion by the entire team. The risk of bias was assessed using the Prediction Model Risk of Bias Assessment Tool (PROBAST) [30], and the extraction form included four domains: participants, predictors, outcomes, and statistical analysis. Results were summarized using descriptive statistics. Quantitative synthesis of the models was not performed.

### Assessment of the feasibility of independent external validation

**Fig. 2:**
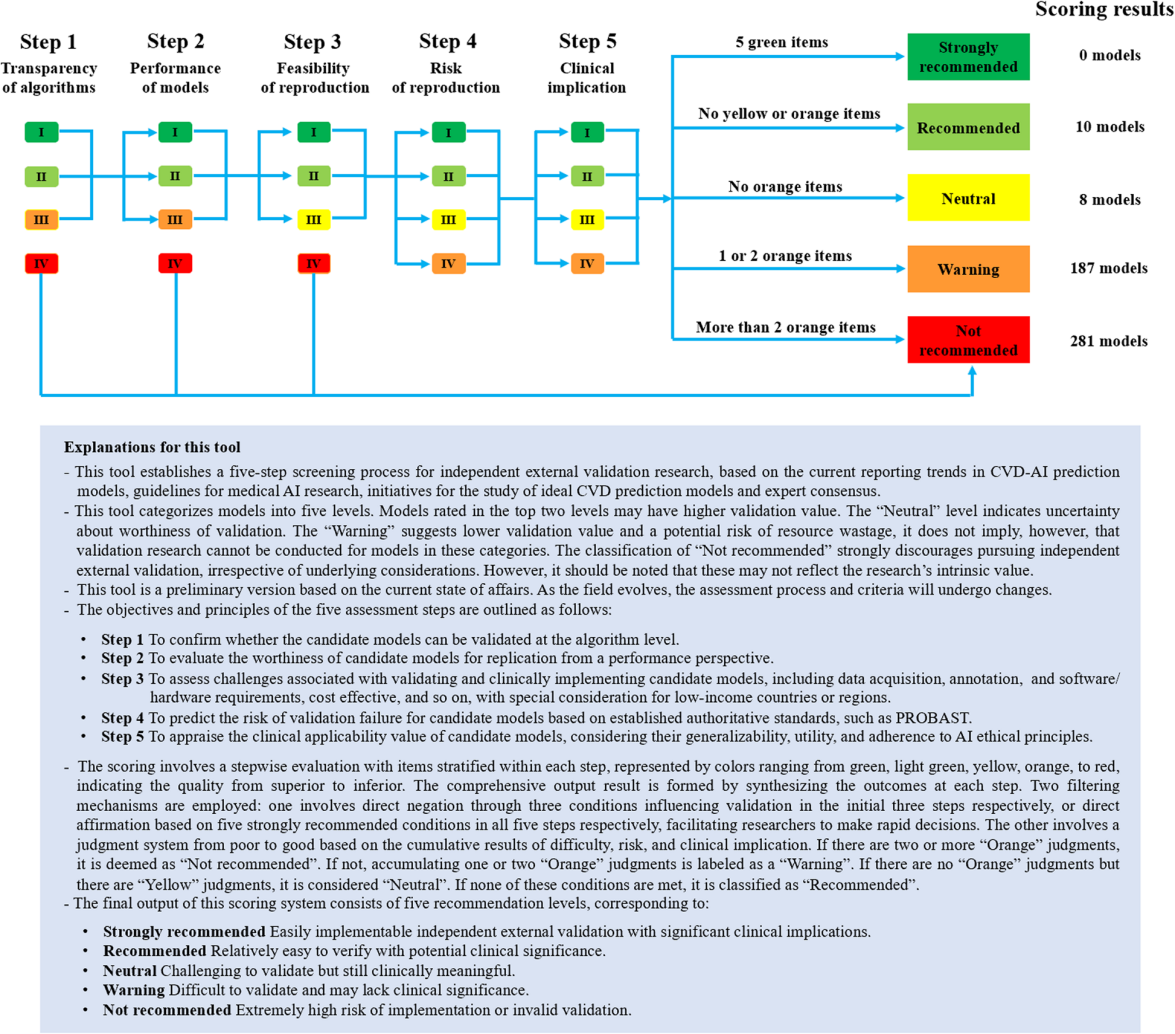
The sketch map of the independent validation score procedure and results

To evaluate the feasibility of independent external validation of each model, we conducted a literature review of existing assessment guidelines or tools in the field of AI/ML medical research (Additional file 2: Text 2 and Fig. S1) and summarized initially candidate items for designing a screening tool. Subsequently, a preliminary plan that weighs screening efficiency and initiatives for the study of ideal CVD prediction models [29] was further discussed and revised by a panel of experts, including clinicians (G-W Z and D-X G), AI experts (T-C J and MG), clinical epidemiologists (ZY), and information technology specialists (WH), among others. Ultimately, a novel scoring system was further developed through consistent feedback of three independent international experts in AI or CVD domains from ExpertScape™ rank and peer recommendations. It is called the independent validation score (IVS), comprising five steps with five score items as follows: transparency of models, performance of models, feasibility of reproduction, risk of reproduction, and clinical implication sequentially. After the five-step scoring process, five grades of feasibility recommendation were set, including “strongly recommended”, “recommended”, “neutral”, “warning”, and “not recommended”. The detailed definitions and rules are shown in Fig. 2 and Table 1.

**Table 1:** The specific evaluation criteria of IVS

Score items	Grade	Specific evaluation criteria	References
**Transparency of algorithms**	I	Post the trained models that can be directly loaded by other researchers for a contiguous independent validation or online/mobile user-friendly calculators that can allow batch processing of participant information (e.g., a prediction software or tool)	∙ APPRAISE-AI [31] ∙ MI-CLAIM [32] ∙ AI-TREE [33]
	II	Apply and report the classic algorithms that can be found in some common tools/platforms OR report complete codes and hyperparameters and required description, allowing independent researchers to run the pipeline end to end	
	III	Report formulas and/or incomplete hyperparameters without required description, leading to difficulties in replication or incomplete reproducibility	
	IV	Incomplete reports that cannot be used for reproduction	
**Performance of models**	I	At least report the discrimination (preferably c-index) and calibration (preferably calibration plot/table) of the model, and the performance index version is clearly reported and index is excellent (e.g., 0.9 < c-index < = 1.0; calibration intercept close to 0 and calibration slope close to 1)	TRIPOD [34] ∙ CHARMS checklist [35] ∙ Official statement [36] ∙ AI-TREE [33] ∙ Expert comment [37]
	II	At least report the discrimination (preferably c-index) and calibration (preferably calibration plot/table) of the model, and the performance index version is clearly reported and index is good (e.g., 0.7 < c-index < = 0.9; calibration intercept deviates moderately from 0, and calibration slope deviates moderately from 1)	
	III	Do not report the discrimination or calibration of the models; OR the performance index version is not clearly reported; OR the value of the index is unknown	
	IV	The model performance is at a low accuracy (e.g., c-index < = 0.7; calibration intercept deviates severely from 0 and calibration slope deviates severely from 1)	
**Feasibility of reproduction**	I	The office-based models without requirement for laboratory and inspection data (also known as non-laboratory models)	∙ Validation and evaluation framework [38] ∙ AI standardization [39] ∙ AI-TREE [33] ∙ MI-CLAIM [32] ∙ CONSORT-AI [40] ∙ MAIC-10 [41] ∙ SR of validity and clinical utility [11] ∙ WHO laboratory-based and non-laboratory models [42] ∙ Laboratory-based and non-laboratory models [43]
	II	The laboratory-based models only requiring routine clinical structured data, which are easy to obtain and do not need secondary operation (e.g., image pre-processing or annotation, etc.)	
	III	Include data derived from unconventional laboratory and inspection, complex gene-related testing, tissue specimen, and other resource-limiting extensive applications, which are hard to obtain or require secondary operation (e.g., labeling)	
	IV	Do not report the variables	
**Risk of reproduction**	I	No domain high risk (evaluated by using PROBAST)	∙ PROBAST [30]
	II	Only one domain is high risk (evaluated by using PROBAST)	
	III	Two domains are high risk (evaluated by using PROBAST)	
	IV	Over two domains are high risk (evaluated by using PROBAST)	
**Clinical implication**	I	Identified novel risk markers or novel risk standards, which will optimize existing clinical preventive strategies and contribute to patient benefit for the general population and major CVDs, similar to classical T-Ms (e.g., Framingham Score)	∙ SR of T-Ms [29] ∙ Biomedical research AI guideline [44] ∙ BS30440 [45] ∙ APPRAISE-AI [31] ∙ Consolidated AI reporting guideline [46] ∙ AI-TREE [33] ∙ SR of validity and clinical utility [11] ∙ Rare CVD [47, 48]
	II	Do not identify novel risk markers or novel risk standards, but enhance the predictive capacity beyond that of existing methods, which may optimize existing clinical preventive measures or offer additional benefits for the non-rare population and non-rare subset of CVDs (more than 1/2000 of the general population)	
	III	Only enhance the predictive capacity beyond that of existing methods, but cannot alter the existing preventive interventions or provide additional benefits for the non-rare population and non-rare subset of CVDs (more than 1/2000 of the general population)	
	IV	Do not enhance the predictive performance beyond that of existing methods OR only target a rare population or subset of CVDs (fewer than 1/2000 of the general population, e.g., infiltrative cardiac diseases), leading to inadequate validation and a lack of clinical utility for a broader population	

## Results

### Study designs and populations

**Fig. 3:**
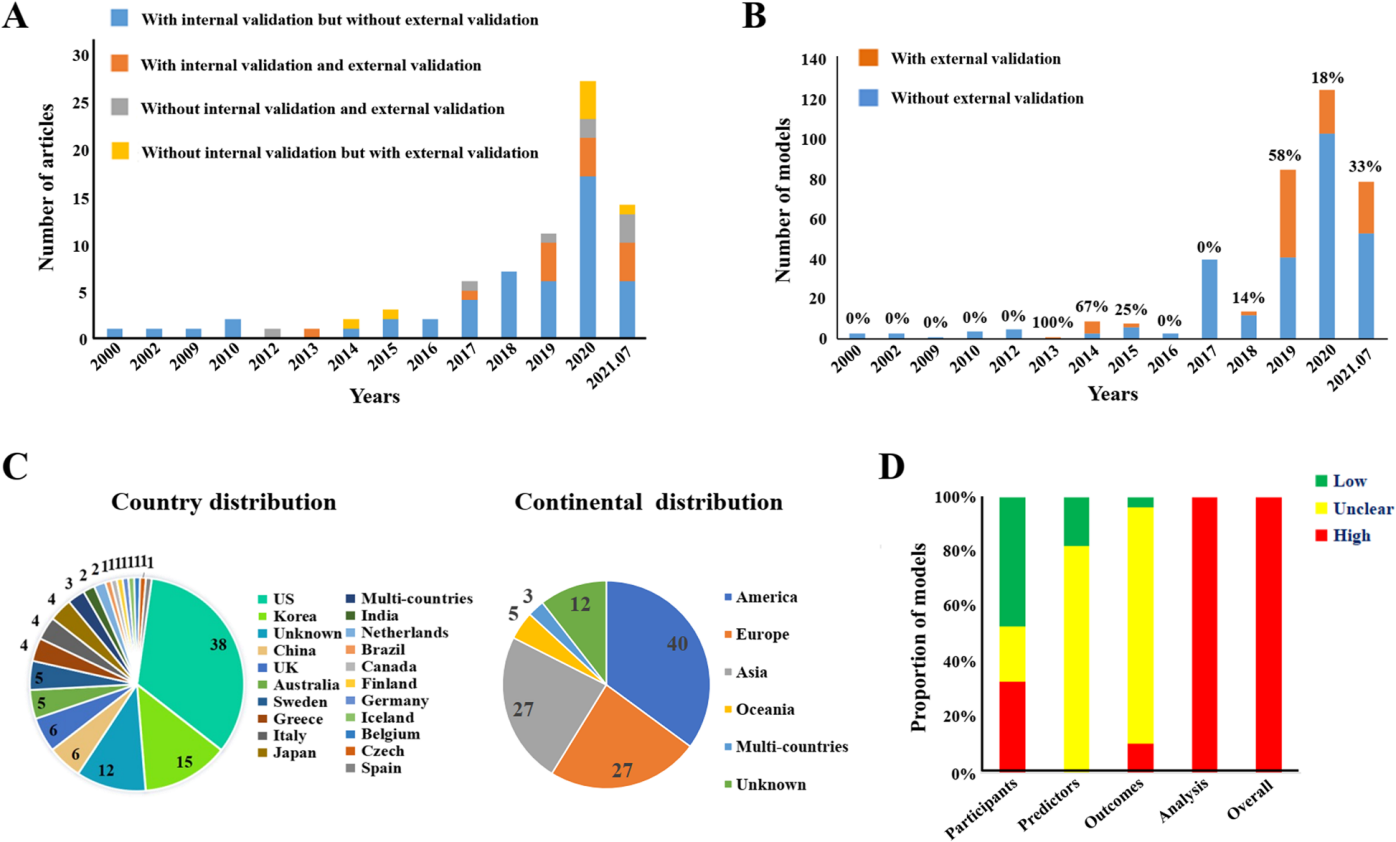
**A** The bar chart of number analysis for articles per year (up to July 31, 2021). **B** The bar chart of number analysis for validated models per year (up to July 31, 2021). **C** The pie graph of papers’ geographical distribution. US, The United States of America; UK, The United Kingdom of Great Britain and Northern Ireland. **D** The bar chart of bias risk analysis with PROBAST

Overall, 79 articles were finally included from 2000 to 2021 (Additional file 2: Table S1) [49–127], with 65 (82.25%) published between 2017 and 2021 (Fig. 3A). In total, 114 cohorts (datasets) were used, with 27 in Europe, 40 in America (mainly the USA), 27 in Asia (mainly Korea), and 5 in Oceania (Australia), 3 multi-country cohorts, but 0 in Africa, as shown in Fig. 3C. A total of 647 different models were identified, including 161 T-Ms excluded from this bibliometric research and 486 AI-Ms involved in the following analysis. Most models were developed using data from 101 trials, and only a minority were from five case–control studies and eight nested case–control studies. All cohort participants were enrolled consecutively.

We included 63 papers focusing on the general population and 16 that addressed subgroups for specific diseases, including type 2 diabetes (*n* = 4, 5%), hypertension (*n* = 4, 5%), and kidney diseases (*n* = 4, 5%). Forty different age ranges were reported across the cohorts, except for 45 cohorts which did not mentioning age range. The two most common ones were 40 to 79 years (*n* = 12, 15%) and 30 to 77 years (*n* = 6, 7%), and the average age ranged from 42 to 78 years. The majority of papers (*n* = 70, 89%) were not sex specific or stratified, with only 24 cohorts having roughly equal proportions of males and females (45–55% females or males in numbers).

### Data sources and research environment

Only a minority of the articles (*n* = 24, 30%) used multiple datasets to develop models as indicated in data source analysis, showing an obvious dominance in single dataset-deriving models. Of all 114 datasets, 42 are multi-centered, and 32 are single-centered; however, 40 databases are unknown. In terms of information collection, only 56 were from electronic health record (EHR), 11 from EHR + questionnaire, 1 from questionnaire + personal interview, and 46 did not clearly mention the data sources. Regarding the issue of missing variables problem, only 5 cohorts clearly described the number of participants with missing variables, whereas 94 cohorts did not mention this value. Fifteen cohorts excluded all participants with missing variables.

In the research environment, the largest number came from the hospital scene (*n* = 44, 39%), followed by community (*n* = 20, 18%), primary health care institutions (*n* = 5, 4%), and hospital scene + primary health care institutions (*n* = 1, 1%). Forty-four cohorts did not state the environment. The study periods ranged from 1965 to 2019, with 57 cohorts reporting the study period, 26 cohorts reporting only the baseline time, and 31 not mentioning the study period.

### Criteria for inclusion and exclusion

Of all 79 articles, only 36 clearly reported the criteria for inclusion, mainly including age restriction, necessary clinical examination and variables, special disease, adequate follow-up time, and number of visits during the period of follow-up. Twenty-two papers did not clearly state the exclusion criteria (Additional file 2: Table S2).

### Predictors

**Fig. 4:**
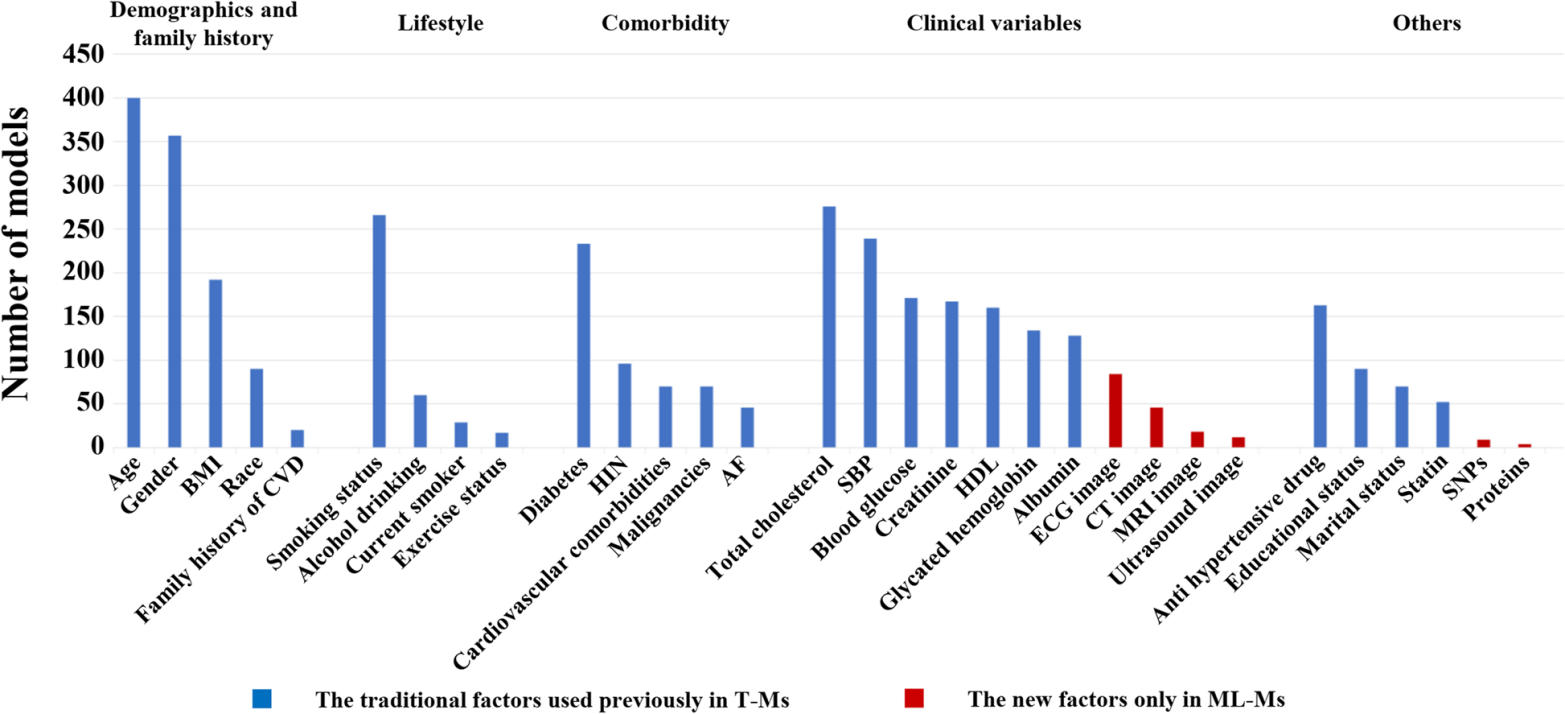
The bar chart of summary and categories of predictors involved in all models

In all AI-Ms, the median number of predictors was 21 (range 5–52,000), with an unquantifiable total number due to a lack of detailed information in individual articles. These predictors were into two types: traditional factors and new-added ones, according to whether they can be addressed by T-Ms. In addition to traditional factors such as age (in 400 models), sex (in 357 models), total cholesterol (in 276 models), and smoking status (in 266 models), several new-added predictors have emerged in AI-Ms, including electrocardiogram (ECG) image (*n* = 84, 17%), ultrasound image (*n* = 44, 9%), magnetic resonance imaging (MRI) image (*n* = 18, 4%), computed tomography (CT) image (*n* = 12, 2%), single nucleotide polymorphisms (SNPs) (*n* = 9, 2%), and proteins (*n* = 4, 1%) as shown in Fig. 4. Further analysis showed that 135 models (30.96%) were built using these new-added data.

### CVD outcomes and measurement method

We found a large variation in predicted outcomes among different models. A total of 42 single endings and 61 combined endings were confirmed in all models. The most common in all 103 endpoints were complete CVD (*n* = 40, 39%) and death (*n* = 16, 16%). However, a considerable heterogeneity was identified in the definitions of these outcomes, such as 19 different definitions for CVD. The main origin of definitions is diverse, including disease codes (ICD9 or ICD10, *n* = 36, 35%), self-report (*n* = 4, 4%), and other international guidelines (*n* = 3, 3%). Additionally, there were 149 models (30.66%) not reporting the definition of the outcomes in 21 papers.

The most common prediction horizons in AI-Ms were 10 (*n* = 107, 22%) and 2.5 years (*n* = 70, 14%) with a range between 1 day and 15 years. Only 25 papers reported the measurement methods for all included outcomes, which primarily comprised clinical records, national institute statistics, questionnaires, and personal interviews. Only 11 articles reported that the outcome measurement was blinded, and two articles explicitly reported not using the blinding method. Other detailed information is summarized in Additional file 2: Table S3.

### Sample size and performance

In total, 4 articles did not report the sample size and 22 articles did not report the number of events. Based on reported data, the number of participants included in AI-Ms ranged between 80 and 3,660,000 (median 4466), and the commonly used order of magnitudes of the number ranged from 1000 to 10,000 (*n* = 44). The ending events occurred ranging from 10 to 152,790 (median 504).

In all the articles (*n* = 79), at least one measure of predictive performance was reported, which was also one of the inclusion criteria for the article in this system review. C index was mainly reported for 482 models. The calibration plot was for 90 models. Sensitivity/recall was for 312 models. Specificity/true negative rate (TNR) was for 209 models. Precision/positive predictive value (PPV) was for 201 models; accuracy was for 199 models; F1 score was for 137 models; Matthews correlation coefficient (MCC) was for 7 models.

### Assessment of algorithms transparency and model reproductivity

Overall, 13 categories of 66 idiographic algorithms were identified based on their operation mechanisms and accepted classification principles. The most frequently applied algorithm in all models is logistic regression (*n* = 74, 15.2%), followed by random forest (*n* = 71, 14.6%) and neural network (*n* = 63, 13.0%) as summarized in Additional file 2: Table S4. Only 26 (39.4%) were used more than 3 times, while 24 (36.4%) appeared only once in all algorithms. In total, 212 models did not report codes, formulas, or hyperparameters, consequently identified as non-reproductive.

### Development models and external validation models

Of the 486 models, 380 were development models and 106 were external validation models (validating 103 development models), as reported in their primary papers. Notably, no independent external validations were found in this field. Additionally, most datasets (*n* = 17, 68%) used for external validation were from the same countries as those used for development models in their primary papers; however, most datasets used for external validation were from different research periods (*n* = 13, 52%) and different settings (*n* = 18, 72%) as those used for development models. The development and external validation of models were conducted by the same investigators in the same article. Our additional exploratory analysis revealed a lower validation propensity in the developed models with new variables (25.24% *vs*. 43.68%, *P* = 0.001) and an AUC < 0.7 (0% *vs*.70.45%, *P* < 0.001), which provide important information for us to build IVS.

### Risk of bias

All models were at high risk of bias (*n* = 486, 100%) according to the assessment using PROBAST, as shown in Fig. 3D. The most common reasons were as follows: 1) inappropriate data sources or inappropriate enrolment strategy in the participant domain (*n* = 161, 33%); 2) not mentioning the definition and measurement of the predictors, or not mentioning whether the predictor’s assessments were blinded to outcome knowledge in the predictor domain (*n* = 401, 83%); 3) inappropriate outcome classification method, outcome definition was not the same for all participants, predictors included in the outcome definition, or the determination of outcomes with the knowledge of predictors in the outcome domain (*n* = 52, 11%); 4) not accounting for the complexities of data, not evaluating the performance of models appropriately, or not accounting for model overfitting and optimism in the statistical analysis domain (*n* = 486, 100%). The details are shown in Additional file 2: Table S5.

### Summary of existing assessment guidelines or tools

Overall, a total 29 of guidelines or tools related to quality assessment or control in the past decade (mainly in the last four years), with 5 for developing quality, 14 for reporting quality, and 10 for both (Additional file 2: Table S6) [11, 30–32, 34, 38, 40, 41, 44–46, 128–145]. In addition to the study design, statistical methods, model performance, risk of bias, AI ethics risk, replicability, as well as clinical implementation, application, and implication in both developing and reporting assessments, the complexity and standardization of data acquisition and processing, required resources (such as software platforms, hardware, or technical professionals), and cost-effectiveness are also focal points in many developing assessments. These provide a core framework for the construction of IVS.

### Independent validation score

Most models were identified as “not recommended” (*n* = 281, 58%) or given a “warning” (*n* = 187, 38%). Only 10 (2%) were classified as “recommended,” and none were identified as “strongly recommended” as revealed by our IVS for all 486 models in Fig. 2. The recommended models are displayed in Additional file 2: Table S7. Insufficient transparency of models contributed the largest number of “not recommended” (*n* = 212), followed in turn by performance (*n* = 56), feasibility of reproduction (*n* = 12), and comprehensive reasons (*n* = 1).

## Discussion

This systematic review is the first to encompass global AI studies of CVD prediction in the general population for more than 20 years, starting from the first article published in 2000 [72]. It presents the current status and broad trends in this field through a comprehensive search and careful selection of studies. We performed an extensive data extraction and thorough analysis of key characteristics in publications, including the predictors, populations, algorithms, performance, and bias. On top of this, we have developed a tool for evaluating replicability and applicability, to screen appropriate AI-Ms for independent external validation, addressing the key issues currently hindering the development of this field. The findings and conclusions are expected to provide references and help for algorithm developers, cohort researchers, healthcare professionals, and policy makers.

### Principal findings

Our results revealed significant inefficiency in external validations and a lack of independent external validation for the existing models, indicating that researchers in the field of AI risk prediction were more inclined to put emphasis on new models developing, instead of validating, although validation is crucial in determining clinical decisions [146]. According to the experience in the field of T-Ms research, these may lead to a large number of useless prediction models, thereby suggesting that more attention should be paid to external validation to avoid research waste and facilitate the translation of high-performing predictive models into clinical practice [147–149]. Based on the facts that most studies used data from only one cohort, we conjecture that limited data source may be one of the main reasons that restrict the implementation of external validations. Therefore, the multi-centers studies, especially multi-countries studies (only three were found in our review), should be encouraged to establish multi-source databases.

It is found that the majority of studies were conducted in Europe and North America, with only a few in the developing countries from Asia and South America, and unfortunately none in Africa. The similar geographical trends have been confirmed in the conventional CVD prediction models through previous literature reviews [29, 150]. However, the prevalence of the CVD is dramatically increasing in those low- or middle-income countries, consequently contributing over three quarters of CVD deaths all over the world and causing great burden to the local medical system [151–154]. Considering the influence of ethnic heterogeneity on the prediction model [155], native AI-Ms tailored to these countries should be developed for local prevention of CVD.

Four classic indexes, age, sex, total cholesterol, and smoking status, were more frequently used in AI-Ms in all presented predictors (some papers not fully representing the used predictors), similar to T-Ms. However, more importantly, the following summary demonstrates that AI-Ms have triggered a profound revolution to predictors owing to its strong data computing capability. First, the median number of predictors in the AI-Ms was approximately 3 times greater than that in T-Ms as collated by Damen et al. [29]. Second, except for the classic predictors (e.g., demographics and family history, lifestyle, and laboratory measures), several new indexes have been involved in AI-Ms, mainly consisting of some multimode data that cannot be recognized and utilized by T-Ms at all (e.g., image factors and gene- or protein-related information). Third, the limitation of data range has been eliminated, as proven by the no fixed age range and sex-specific equation for the development of AI-Ms, which were important concerns in classic T-Ms. Fourth, AI models allow data re-input and utility. Researchers gathered data many times in the follow-up procedure in recurrent neural network (RNN) models, and these time series data were used to retrain the AI-Ms for further improvement of performance [55, 112]. Another interesting improvement is that the screening of predictors could be executed automatically by AI instead of classic log calculation [50, 52].

The systematic review of specific models is imperative for the head-to-head comparison of these models and the design of the relevant clinical trials [156, 157]. Our analysis of report quality was performed through reference to the TRIPOD statement and CHARMS-CHECKLIST, to inform readers regarding how the study was carried out [158]. Worryingly, we found that many articles did not report important research information, which not only significantly restrict the readability of articles largely but also may lead to the unwarranted neglect for the previous evidence through subsequent researches [159–162]. Therefore, we have to strongly recommend that each study should upload a statement of TRIPOD or upcoming TRIPOD-AI designed specifically for AI prediction models when the manuscripts were submitted [12, 163, 164].

According to PROBAST, a common evaluation method of risk of bias for traditional prediction models [165], all included AI-Ms were judged as high risk in our summary, mainly owing to ignorance or failure to report competing risk in the item of statistical analysis. Similar trends of high risk have been confirmed in many previous systematic reviews regarding AI-Ms for other diseases, although there are some differences in specific reasons, which involved more frequently sample size, calibration, missing data handling, and so on [12, 166–168]. This could potentially be another significant constraint on the independent external validation of models, in addition to the various issues mentioned earlier, which currently hinder the widespread adoption of AI-Ms for CVD clinical practice. Therefore, it is strongly suggested again that more attention should be focussed on statistical analysis, not only for authors in the research and writing process, but also for reviewers and editors during review and publication. Meanwhile, these widely high-risk judgment ratios prompt us to raise question whether the current criteria are too harsh for AI-Ms, because it is unclear whether some algorithms may offset competing risk due to their “black box” effect, and it should not be ignored that the classic method of EPV may not be suitable for the sample size calculation in some ML algorithms owing to their specific operation mechanism [169–171].

Best practice guidance and specific pathways for the translation of AI-healthcare research into routine clinical applications have been developed. Holmes et al. summarized the AI-TREE criteria [33], while Banerjee et al. created a pragmatic framework for assessing the validity and clinical utility of ML studies [11]. Building on this prior work and the experiences reported in studies involving AI risk prediction models for various diseases [75, 172–174], our insights gained during the validation process of existing AI models, as well as a combination of summary of existing AI research assessment guidelines or tools and experts’ suggestions, we have developed an IVS for screening independent external validation models. This tool is primarily intended for researchers involved in the validation process rather than developers during the implementation phase. In this scoring system, in addition to the two recognized criteria of transparency and risk assessment, the performance and clinical implication were included to determine their suitability for independent external validation, which to some extent, align with factors typically considered during the model development process, such as impact, cost-effectiveness, and AI-ethics [11, 33]. In assessing performance, we opted for the two most widely reported and strongly recommended indices for discrimination and calibration, namely the c index and calibration plot/table, instead of specificity or sensitivity, as they are not recommended by the TRIPOD and checklist guidelines [34, 35, 158]. Furthermore, the consistency of retrospective validation datasets and the challenges in acquiring prospective study data are key factors influencing external validation [75, 172–174], especially in the case of factors like imaging, biomarkers, genomics, which may also encounter issues such as lack of standardization and biased reporting [33]. Building upon the WHO's principles of model utility [42], the acquisition and handling of laboratory-based and emerging multimodal predictive factors’ acquisition and handling are essential assessment components in evaluating the feasibility of independent external validation.

Our IVS results have indicated that more than 95% of the models may not be suitable for independent external validation by other researchers, and as a result, may not provide any useful help for the following clinical application. Therefore, it is rather reasonable to explain why there have been no independent external validation researches in the field of CVD-AI prediction for over 20 years. In addition to the problem of model transparency, the following other four reasons also are considered to account for irreproducibility of the models, including increased difficulty in parameter acquisition and processing, uncertain expected performance, and low reliability owing to high risk. Therefore, it is strongly suggested that the assessment of model replicability should be performed in the process of project research, and a statement of IVS should be reported at the time of submission. However, even after screening, it is still necessary to comprehensively consider other factors, such as unquantifiable AI ethics issues, due to the emphasis on assessing technical feasibility and impact in the scoring system. It is also important to emphasize that the current scoring system remains theoretical and requires practical validation and adjustment, necessitating input and refinement from numerous scholars.

### Challenges and opportunities

Despite over 20 years of development, the AI field of CVD prediction experienced a surge of articles in the past 5 years, accompanied by the aforementioned phenomena regarding the emphasis on development but validation, no independent validation studies, and a large number of new algorithms studied only once. This field has been concluded as being in an early stage of development, similar to the traditional Framingham model from the 1970s to 1990s [175, 176]. Different from T-Ms, however, the AI ones are quite hard to comprehend and implement for clinical researchers owing to their complexity and “black box”. Meanwhile, there appear continually new algorithms or new combinations of the existing (such as model averaging and stacked regressions), even there may be rather different ranking indexes in the same algorithm [160, 177]. Therefore, it is reasonable to speculate that new exploratory research will continue to dominate for the foreseeable future, which may be the inherent demand for this field, although the external validation of existing models was necessary to avoid research waste, as advocated strongly by many researchers [10, 11, 164].

Several pivotal problems limiting the development of this field still require to be emphasized again. First, the solution to study design and reporting defects, including insufficient external validation, geographical imbalance, inappropriate data sources, and deficiency in algorithm details, largely depends on improving scientific research consciousness and level of all researchers in this industry, which is a gradual process, and thereby uneven development and research waste will be difficult to stop in a short time. Second, another grim situation is how to improve model intelligibility, reproducibility, and replicability, which may far outweigh our understanding concluded in the studies of T-Ms, although some researchers have been making great efforts to explore underlying mechanisms of AI operation, with the increasingly intense expectations of a revolutionary breakthrough as soon as possible [178]. Additionally, it is urgent to establish an integral system of quality control and performance evaluation for the studies in this field. However, this requires a gradual development process, although the World Health Organization (WHO) and International Telecommunication Union (ITU) have established a Focus Group on Artificial Intelligence for Health (FG-AI4H), which has begun shaping guidelines and benchmarking process for health AI models through an international, independent, and standard evaluation framework to guide and standardize the industry development [179].

In addition to the challenges posed by the “black box” issue leading to non-interpretable problems, biases and fairness, technical safety, preservation of human autonomy, privacy, and data security are significant AI ethics concerns within this field [20, 180]. The development of trustworthy AI in healthcare has become a crucial responsibility worldwide [181]. For instance, the European Commission has enacted both the “Ethics Guidelines for Trustworthy AI” and the “Artificial Intelligence Act” [182, 183]. Similarly, in the USA, the creation of the National AI Initiative Office aims to promote the development and utilization of trustworthy AI in both the public and private sectors [184]. Although the articles in this review have devoted limited discussion to these topics, it is essential to note that the aforementioned aspects (including improvement of model transparency and interpretability, reduction in bias risk, enhancement of reproducibility, as well as placing additional emphasis on data and privacy protection), in addition to their scientific research roles, also play a crucial role in addressing AI ethics concerns. These efforts are beneficial for alleviating public concerns about AI ethics issues related to predictive models, thereby increasing trust and acceptance of the models. These aspects improve the balance between AI-assisted decision-making and the preservation of human autonomy, facilitating the clinical application and dissemination of the models. Therefore, we strongly recommend that AI ethics considerations be thoroughly integrated into the model development and validation processes.

For AI intervention studies, the relatively excellent guidelines for the design, implementation, reporting, and evaluation have been developed by the EQUATOR-network, including STARD-AI, CONSORT-AI, and SPIRIT-AI, as well as different scientific journals and associations [139, 142, 185–187]. These guidelines will also serve as a roadmap for the development of predictive AI. In practice, Banerjee et al. have designed a seven-domain, AI-specific checklist based on AHA QUADAS-2 CHARMS PROGRESS TRIPOD AI-TREE and Christodoulou, to evaluate the clinical utility and validity of predictive AI algorithms [11]. Oala et al. are building a tool of AI algorithm auditing and quality control for more effective and reliable application of ML systems in healthcare, helping to manage dynamic workflows that may vary through used case and ML technology [188]. Collins et al. have begun to develop TRIPOD-AI and PROBAST-AI for AI prediction models [21, 163, 164]. Additionally, based on the results of our IVS analysis, we are planning an independent external validation study with multiple datasets to fill the gap in AI field of CVD prediction. These will be expected to propel this field into a new and mature stage of development.

### Recommendations

Despite the increasing recommendations by healthcare providers and policymakers for the use of prediction models within clinical practice guidelines to inform decision-making at various stages in the clinical pathway [161, 189], we still suggest that experts in this field should put more emphasis on establishment and implementation of scientific research guidelines, for example, promoting ML4H supervision and management for AI prediction models [188]. Additionally, referring to the requirements for intervention AI statement, some AI-relevant information should be added into TRIPOD-AI, such as algorithm formulas, hyperparameter tuning, predictive performance, interpretability, sample size determination, and so on [186, 190]. Certain items in PROBAST need to be modified for AI prediction models, especially 2.3, 4.1, and 4.9, due to inappropriate standards or nonexistent coefficients in some algorithms. Items 4.6–4.8 should be renegotiated on the premise of fully considering the algorithm characteristics. Furthermore, algorithm auditing, overfitting control, sample size calculation, and identification of variables in image data should be added into PROBAST-AI.

In light of studies on conventional models, a greater responsibility falls upon AI algorithm developers, which include improving the transparency in reporting to facilitate model reproduction, and heightening the comprehensibility and enforceability of algorithms to users for wider clinical practice [191]. Furthermore, we should improve the transparency of reporting not only at the time of publication but also in the process of pre-submission, reviewing, or post-publication stages. Meanwhile, editors and reviewers should also play a key role in improving the quality of reporting.

### Study limitations

The systematic review has several limitations. Firstly, similar to other studies [10, 11, 29], the papers not in English, without available full text, or published in other forms (for example, conferences, workshops, news reports, even the unpublished) were also excluded in our review, which may lead to an underestimation of the number of models and an imbalance in geographical contribution as mentioned above. Second, the potential impact of AI on healthcare might still be overestimated during the present procedure of retrospective literature analysis, owing to unavoidable publication bias and reporting bias, despite some measures that have been performed to reduce the omission of included literature [11, 192]. Furthermore, we did not evaluate the clinical usefulness aspects such as net benefit or impact study [159, 193, 194], which are outside our scope and require further investigation.

## Conclusions

In summary, AI has triggered a promising digital revolution for CVD risk prediction. However, this field is still in its early stage, characterized by geographical imbalance, low reproducibility, a lack of independent external validation, a high risk of bias, a low standard-reaching rate of report quality, and an imperfect evaluation system. Additionally, the IVS method we designed may provide a practical tool for assessing model replicability. It is expected to contribute to independent external validation research and subsequent extensive clinical application. The development of AI CVD risk prediction may depend largely on the collaborative efforts of researchers, health policymakers, editors, reviewers, as well as quality controllers.

### Supplementary Information

**Additional file 1.** PRISMA 2020 Checklist.

**Additional file 2:** **Text 1.** Search strategies for AI-Ms of CVD prediction. **Table S1.** Characteristics of the included studies. **Table S2.** Inclusion and exclusion criteria of the included studies. **Table S3.** The definition and measurement of outcomes. **Table S4.** The counting and characteristics of algorithms. **Table S5.** Risk of bias assessment of prediction models. **Text 2.** Search strategies of AI/ML assessment guidelines or tools. **Fig. S1.** The flow diagram for literature search in the assessment guidelines or tools in the field of medical AI/ML research. **Table S6.** The characteristics of assessment guidelines or tools in the field of medical AI/ML research. **Table S7.** The characteristics of 10 recommended models.

## Data Availability

The datasets generated and/or analyzed during the current study are available in PubMed, Web of Science, Embase, and IEEE library up to July 2021.

## Abbreviations

ACC/AHA: American College of Cardiology/American Heart Association
AI-Ms: Artificial intelligence models
CT: Computed tomography
CVD: Cardiovascular disease
DL: Deep learning
ECG: Electrocardiogram
EHR: Electronic health record
ESC: European Society of Cardiology
FG-AI4H: Focus Group on Artificial Intelligence for Health
ITU: International Telecommunication Union
IVS: Independent validation score
MCC: Mattews correlation coefficient
ML: Machine learning
MRI: Magnetic resonance imaging
PPV: Positive predictive value
PROBAST: Prediction risk of bias assessment tool
RNN: Recurrent neural network
SNPs: Single nucleotide polymorphisms
T-Ms: Traditional models
TNR: True negative rate
WHO: World Health Organization
