# Supplementary material for: Artificial intelligence in the risk prediction models of cardiovascular disease and development of an independent validation screening tool: a systematic review
Source: BMC Med. 2024 Feb 5;22:56. doi: 10.1186/s12916-024-03273-7 (PMC10845808; doi:10.1186/s12916-024-03273-7)
Supplement: Supplementary file 2 — Additional file 2: Text 1. Search strategies for AI-Ms of CVD prediction. Table S1. Characteristics of the included studies. Table S2. Inclusion and exclusion criteria of the included studies. Table S3. The definition and measurement of outcomes. Table S4. The counting and characteristics of algorithms. Table S5. Risk of bias assessment of prediction models. Text 2. Search strategies of AI/ML assessment guidelines or tools. Fig. S1. The flow diagram for literature search in the assessment guidelines or tools in the field of medical AI/ML research. Table S6. The characteristics of assessment guidelines or tools in the field of medical AI/ML research. Table S7. The characteristics of 10 recommended models. [file 12916_2024_3273_MOESM2_ESM.doc]

**Text 1 Search strategies for AI-Ms of CVD prediction**

**Search strategy PubMed**

(Heart failure[MeSH Terms] OR Atrial fibrillation[MeSH Terms] OR Acute coronary syndrome[MeSH Terms] OR Cardiovascular diseases[MeSH Terms] OR Myocardial infarction[MeSH Terms] OR coronary Artery disease[MeSH Terms] OR stroke[MeSH Terms] OR cerebrovascular disorders[MeSH Terms] OR heart disease[MeSH Terms] OR arrhythmias, Cardiac[MeSH Terms] OR Angina, Pectoris [MeSH Terms]OR myocardial ischemia[MeSH Terms] OR All-cause mortality[MeSH Terms] OR Coronary artery bypass [MeSH Terms] OR Percutaneous transluminal coronary angioplasty[MeSH Terms] OR Heart failure[Text Word] OR Atrial fibrillation[Text Word]OR Acute coronary syndrome[Text Word] OR Cardiovascular diseases[Text Word] OR Myocardial infarction[Text Word] OR coronary Artery disease[Text Word] OR stroke[Text Word] OR cerebrovascular disorders[Text Word] OR heart disease[Text Word] OR arrhythmias, Cardiac[Text Word] OR Angina, Pectoris [Text Word]OR myocardial ischemia[Text Word] OR All-cause mortality[Text Word] OR Coronary artery bypass [Text Word] OR Percutaneous transluminal coronary angioplasty[Text Word])

AND

(Machine learning [MeSH Terms] OR Artificial intelligence [MeSH Terms] OR Unsupervised [Text Word] OR Supervised [Text Word] OR Machine learning [Text Word] OR Artificial intelligence [Text Word])

AND

(Risk [MeSH Terms] OR Mortality [MeSH Terms] OR Risk prediction [Title/Abstract] OR Prediction [Text Word])

**Search strategy Web of science**

TS=(Heart failure OR Atrial fibrillation OR Acute coronary syndrome OR Cardiovascular diseases OR Myocardial infarction OR coronary Artery disease OR stroke OR cerebrovascular disorders OR heart disease OR arrhythmias, Cardiac OR Angina, Pectoris OR myocardial ischemia OR All-cause mortality OR Coronary artery bypass OR Percutaneous transluminal coronary angioplasty)

AND

TS= (Machine learning OR Artificial intelligence OR Unsupervised OR Supervised)

AND

TS=(Risk OR Mortality OR Risk prediction OR Prediction)

**Search strategy IEEE**

All Metadata (Heart failure OR Atrial fibrillation OR Acute coronary syndrome OR Cardiovascular diseases OR Myocardial infarction OR coronary Artery disease OR stroke OR cerebrovascular disorders OR heart disease OR arrhythmias, Cardiac OR Angina, Pectoris OR myocardial ischemia OR All-cause mortality OR Coronary artery bypass OR Percutaneous transluminal coronary angioplasty)

AND

All Metadata (Machine learning OR Artificial intelligence OR Unsupervised OR Supervised OR Mortality prediction OR Risk prediction OR Prediction)

**Search strategy Embase**

1. MESH (Heart failure OR Atrial fibrillation OR Acute coronary syndrome OR Cardiovascular disease OR heart infarction OR coronary Artery disease OR cerebrovascular accident OR cerebrovascular disease OR heart disease OR heart arrhythmia OR angina pectoris OR heart muscle ischemia OR all-cause mortality OR coronary artery bypass graft OR transluminal coronary angioplasty)

2. TI AB KW (Heart failure OR Atrial fibrillation OR Acute coronary syndrome OR Cardiovascular disease OR heart infarction OR coronary Artery disease OR cerebrovascular accident OR cerebrovascular disease OR heart disease OR heart arrhythmia OR angina pectoris OR heart muscle ischemia OR all-cause mortality OR coronary artery bypass graft OR transluminal coronary angioplasty)

3. #1 OR #2

4. MESH (Machine learning OR Artificial intelligence)

5. TI AB KW (Unsupervised OR Supervised OR Machine learning OR Artificial intelligence)

6. #4 OR #5

7. MESH (Risk OR Mortality)

8. TI AB KW (Risk prediction OR Prediction)

9. #7 OR #8

10. #3 AND #6 AND #9

**Table S1. Characteristics of the included studies**

| **Reference list of included studies** | **First author** | **PY** | **Population** | **IV** | **EV** |
| --- | --- | --- | --- | --- | --- |
| Added Value of a Resting ECG Neural Network That Predicts Cardiovascular Mortality | Marco V. Perez | 2009 | General | Y | N |
| Machine learning based risk prediction model for asymptomatic individuals who underwent coronary artery calcium score: Comparison with traditional risk prediction approaches | Donghee Han | 2019 | General | Y | Y |
| Machine learning and atherosclerotic cardiovascular disease risk prediction in a multi-ethnic population | Andrew Ward | 2020 | General | Y | N |
| Machine Learning Adds to Clinical and CAC Assessments in Predicting10-Year CHD and CVD Deaths | Rine Nakanishi | 2020 | General | Y | N |
| Machine learning outperforms ACC/AHA CVD risk calculator in MESA | Ioannis A. Kakadiaris | 2018 | General | Y | Y |
| Statistics and Deep Belief Network-Based Cardiovascular Risk Prediction | Jaekwon Kim | 2017 | General | Y | N |
| Development and External Validation of a Deep Learning Algorithm for Prognostication of Cardiovascular Outcomes | In-Jeong Cho | 2020 | General | Y | Y |
| Cardiovascular Event Prediction by Machine Learning: The Multi-Ethnic Study of Atherosclerosis | Bharath Ambale-Venkatesh | 2017 | General | Y | N |
| Cardiovascular disease risk prediction using automated machine learning: A prospective study of 423,604 UK Biobank participants | Ahmed M. Alaa | 2019 | General | Y | N |
| Consistency of variety of machine learning and statistical models in predicting clinical risks of individual patients: longitudinal cohort study using cardiovascular disease as exemplar | Yan Li | 2020 | General | Y | N |
| Can machine-learning improve cardiovascular risk prediction using routine clinical data? | Stephen F. Weng | 2017 | General | Y | N |
| Machine learning to predict the long-term risk of myocardial infarction and cardiac death based on clinical risk, coronary calcium, and epicardial adipose tissue: a prospective study | Frederic Commandeur | 2020 | General | Y | N |
| Non-invasive modelling methodology for the diagnosis of coronary artery disease using fuzzy cognitive maps | Ioannis D. Apostolopoulos | 2020 | General | Y | N |
| Blood-Based Biomarkers for Predicting the Risk for Five-Year Incident Coronary Heart Disease in the Framingham Heart Study via Machine Learning | Meeshanthini V. Dogan | 2018 | General | Y | N |
| Accurate Prediction of Coronary Heart Disease for Patients With Hypertension From Electronic Health Records With Big Data and Machine-Learning Methods: Model Development and Performance Evaluation | Zhenzhen Du | 2020 | Hypertension | Y | N |
| A novel neural-inspired learning algorithm with application to clinical risk prediction | Darwin Tay | 2015 | General | Y | N |
| Implications of Cardiovascular Disease Risk Assessment Using the WHO/ISH Risk Prediction Charts in Rural India | Arvind Raghu | 2015 | General | Y | N |
| Evaluation of Risk Prediction Models of Atrial Fibrillation (From the Multi-Ethnic Study of Atherosclerosis [MESA]) | Joshua D. Bundy | 2020 | General | Y | N |
| Development of Health Parameter Model for Risk Prediction of CVD Using SVM | P.Unnikrishnan | 2016 | General | Y | N |
| In Search of an Optimal Subset of ECG Features to Augment the Diagnosis of Acute Coronary Syndrome at the Emergency Department | Zeineb Bouzid | 2021 | Chest pain | Y | Y |
| Machine learning-based prediction of acute coronary syndrome using only the pre-hospital 12-lead electrocardiogram | Salah Al-Zaiti | 2020 | Chest pain | Y | Y |
| Assessing cardiovascular risks from a mid-thigh ct image: a tree-based machine learning approach using radiodensitometric distributions | Carlo Ricciardi | 2020 | General | Y | N |
| Genetic Variants and Their Interactions in the Prediction of Increased Pre-Clinical Carotid Atherosclerosis: The Cardiovascular Risk in Young Finns Study | Sebastian Okser | 2010 | General | Y | N |
| Models to predict cardiovascular risk: comparison of CART, Multilayer perceptron and logistic regression | Gilles Chatellier | 2000 | General | Y | N |
| Prediction Modeling Using EHR Data | Jionglin Wu | 2010 | General | Y | N |
| Prediction of risk of coronary events in middle-aged men in the Prospective Cardiovascular Münster Study (PROCAM) using neural networks | Reinhard Voss | 2002 | General | Y | N |
| Development and Validation of Machine Learning–Based Race-Specific Models to Predict 10-Year Risk of Heart Failure | Matthew W. Segar | 2021 | General | Y | Y |
| Long-Term Exposure to Elevated Systolic Blood Pressure in Predicting Incident Cardiovascular Disease: Evidence From Large-Scale Routine Electronic Health Records | Jose Roberto Ayala Solares | 2019 | Hypertension | Y | N |
| Use of machine-learning algorithms to determine features of systolic blood pressure variability that predict poor outcomes in hypertensive patients | Ronilda C. Lacson | 2018 | Hypertension | Y | N |
| A New Hybrid XGBSVM Model: Application for Hypertensive Heart Disease | Wenbing Chang | 2019 | Hypertension | Y | N |
| Clinical Implication of Machine Learning in Predicting the Occurrence of Cardiovascular Disease Using Big Data (Nationwide Cohort Data in Korea) | Gihun Joo | 2020 | General | Y | N |
| Novel Approaches for Predicting Risk Factors of Atherosclerosis | V. Sree Hari Rao | 2020 | General | N | Y |
| Role of artificial intelligence in cardiovascular risk prediction and outcomes: comparison of machine‑learning and conventional statistical approaches for the analysis of carotid ultrasound features and intra‑plaque neovascularization | Amer M. Johri | 2021 | General | Y | N |
| Stroke risk prediction using machine learning: a prospective cohort study of 0.5 million Chinese adults | Matthew Chun | 2021 | General | Y | N |
| Real-time AI prediction for major adverse cardiac events in emergency department patients with chest pain | Pei-I Zhang | 2020 | General | Y | N |
| Bioimpedance and New-Onset Heart Failure: A Longitudinal Study of >500 000 Individuals From the General Population | Daniel Lindholm | 2018 | General | Y | N |
| Comparison of machine learning approaches towards assessing the risk of developing cardiovascular disease as a long-term diabetes complication | Konstantia Zarkogianni | 2017 | Type 2 Diabetes | Y | N |
| Distinct Dimensions of Kidney Health and Risk of Cardiovascular Disease, Heart Failure, and Mortality | Alexandra K. Lee | 2019 | Chronic kidney disease | N | N |
| Development and Validation of a Clinical Risk-Assessment Tool Predictive of All-Cause Mortality | Ghalib A. Bello | 2015 | General | N | Y |
| Predicting Cardiovascular Risk Using Social Media Data: Performance Evaluation of Machine-Learning Models | Anietie U Andy | 2021 | General | Y | N |
| Comparative assessment of statistical and machine learning techniques towards estimating the risk of developing type 2 diabetes and cardiovascular complications | Kalliopi Dalakleidi | 2017 | Type 2 Diabetes | N | N |
| Pre‑existing and machine learning‑based models for cardiovascular risk prediction | Sang-Yeong Cho | 2021 | General | N | N |
| Metabolic syndrome, fatty liver, and artificial intelligence-based epicardial adipose tissue measures predict long-term risk of cardiac events: a prospective study | Andrew Lin | 2021 | General | N | N |
| Improved long-term prognostic value of coronary CT angiography-derived plaque measures and clinical parameters on adverse cardiac outcome using machine learning | Christian Tesche | 2021 | General | Y | N |
| A hybrid recurrent neural network-logistic chaos-based whale optimization framework for heart disease prediction with electronic health records | P.Priyanga | 2020 | General | Y | N |
| An Efficient Convolutional Neural Network for Coronary Heart Disease Prediction | Aniruddha Dutta Conceptualization | 2020 | General | N | Y |
| Assessment of a Machine Learning Model Applied to Harmonized Electronic Health Record Data for the Prediction of Incident Atrial Fibrillation | Premanand Tiwari | 2020 | General | Y | N |
| Cardiovascular Disease Prediction by Machine Learning Algorithms Based on Cytokines in Kazakhs of China | Yunxing Jiang | 2021 | General | Y | N |
| Cardiovascular/stroke risk predictive calculators: a comparison between statistical and machine learning models | Ankush Jamthikar | 2020 | General | N | N |
| Development and Validation of a Risk Stratification Model Using Disease Severity Hierarchy for Mortality or Major Cardiovascular Event | Che Ngufor | 2020 | General | N | Y |
| Electronic Health Record–Based Prediction of 1-Year Risk of Incident Cardiac Dysrhythmia: Prospective Case-Finding Algorithm Development and Validation Study | Yaqi Zhang | 2021 | General | Y | Y |
| Electronic Medical Record Risk Modeling of Cardiovascular Outcomes Among Patients with Type 2 Diabetes | Dongzhe Hong | 2021 | Type 2 Diabetes | N | N |
| New computer model for prediction of individual 10-year mortality on the basis of conventional atherosclerotic risk factors | Kinuka Ogata | 2013 | General | Y | Y |
| Near-term prediction of sudden cardiac death in older hemodialysis patients using electronic health records | Benjamin A. Goldstein | 2014 | Hemodialysis | N | Y |
| Artificial neural networks versus proportional hazards Cox models to predict 45-year all-cause mortality in the Italian Rural Areas of the Seven Countries Study | Paolo Emilio Puddu | 2012 | General | N | N |
| Prognostic Value of Combined Clinical and Myocardial Perfusion Imaging Data Using Machine Learning | Julian Betancur | 2018 | General | Y | N |
| Machine learning for prediction of all-cause mortality in patients with suspected coronary artery disease: a 5-year multicentre prospective registry analysis | Manish Motwani | 2016 | General | Y | N |
| Machine Learning for Risk Prediction of Acute Coronary Syndrome | Jacob P. VanHouten | 2014 | General | Y | N |
| Machine Learning Improves Cardiovascular Risk Definition for Young, Asymptomatic Individuals | Fátima Sánchez-Cabo | 2020 | General | Y | Y |
| Stroke Prediction with Machine Learning Methods among Older Chinese | Yafei Wu | 2020 | General | Y | N |
| Artificial Intelligence–Electrocardiography to Predict Incident Atrial Fibrillation | Georgios Christopoulos | 2020 | General | N | N |
| Improved cardiovascular risk prediction using targeted plasma proteomics in primary prevention | Renate M. Hoogeveen | 2020 | General | Y | Y |
| Machine learning provides evidence that stroke risk is not linear: The non-linear Framingham stroke risk score | Agni Orfanoudaki | 2019 | General | Y | Y |
| A study of generalizability of recurrent neural network-based predictive models for heart failure onset risk using a large and heterogeneous EHR data set | Laila R Bekhet | 2018 | General | Y | N |
| Multiplex proteomics for prediction of major cardiovascular events in type 2 diabetes | Christoph Nowak | 2018 | Type 2 diabetes | Y | N |
| Machine learning methodologies versus cardiovascular risk scores, in predicting disease risk | Alexandros C. Dimopoulos | 2018 | General | Y | N |
| Learning from Longitudinal Data in Electronic Health Record and Genetic Data to Improve Cardiovascular Event Prediction | Juan Zhao | 2019 | General | Y | N |
| Comparison of risk models for mortality and cardiovascular events between machine learning and conventional logistic regression analysis | Shinya Suzuki | 2019 | General | Y | N |
| A machine learning-based approach for predicting the outbreak of cardiovascular diseases in patients on dialysis | Sabrina Mezzatesta | 2019 | Hemodialysis | Y | Y |
| Development and verification of prediction models for preventing cardiovascular diseases | Ji Min Sung | 2019 | General | Y | Y |
| Machine learning to predict cardiovascular risk | Jose A. Quesada | 2019 | Hypertension, dyslipidemia, or diabetes | Y | N |
| Development, validation, and proof-of-concept implementation of a two-year risk prediction model for undiagnosed atrial fibrillation using common electronic health data (UNAFIED) | Randall W. Grout | 2021 | General | N | Y |
| Predicting Australian Adults at High Risk of Cardiovascular Disease Mortality Using Standard Risk Factors and Machine Learning | Shelda Sajeev | 2021 | General | Y | Y |
| Improved cardiovascular risk prediction in patients with end-stage renal disease on hemodialysis using machine learning modeling and circulating microribonucleic acids | David de Gonzalo-Calvo | 2020 | Hemodialysis | Y | N |
| Long-term PM(2.5) exposure and the clinical application of machine learning for predicting incident atrial fibrillation | In-Soo Kim | 2020 | General | Y | N |
| Machine Learning Based Risk Prediction for Major Adverse Cardiovascular Events | Michael SCHREMPF | 2021 | General | Y | N |
| Logistic regression was as good as machine learning for predicting major chronic diseases | Simon Nusinovici | 2020 | General | Y | N |
| A machine-learning approach to cardiovascular risk prediction in psoriatic arthritis | Luca Navarini | 2020 | Psoriatic arthritis | N | Y |
| Prediction of incident myocardial infarction using machine learning applied to harmonized electronic health record data | Divneet Mandair | 2020 | General | Y | N |

**PY:** Publication year; **IV**: Internal validation; **EV**: External validation.

**Table S2. Inclusion and exclusion criteria of the included studies**

| **First author** | **Publication year** | **Inclusion criteria** | **Exclusion criteria** |
| --- | --- | --- | --- |
| Marco V. Perez | 2009 | Clinical treadmill testing | NA |
| Donghee Han | 2019 | NA | NA |
| Andrew Ward | 2020 | Aged 18 years or older | Prior CVD and on statins, with <5 years of ASCVD event-free follow-up |
| Rine Nakanishi | 2020 | Aged 18 years or older; available CAC scans | Symptoms, history of CHD |
| Ioannis A. Kakadiaris | 2018 | NA | cardiovascular issues, incomplete variable |
| Jaekwon Kim | 2017 | NA | NA |
| In-Jeong Cho | 2020 | Health examinations at least 2 times at baseline | CVD or death |
| Bharath Ambale-Venkatesh | 2017 | NA | Clinical cardiovascular disease |
| Ahmed M. Alaa | 2019 | NA | Coronary heart disease, other heart disease, stroke, transient ischaemic attack, peripheral arterial disease, or cardiovascular surgery |
| Yan Li | 2020 | NA | CVD, prescription for a statin |
| Stephen F. Weng | 2017 | NA | CVD, lipid disorder prescribed lipid lowering drugs |
| Frederic Commandeur | 2020 | age<80 years | Cardiac or cerebrovascular disease or chest pain terminal malignancy, cirrhosis, or end-stage renal disease, pregnancy |
| Ioannis D. Apostolopoulos | 2020 | NA | Factor of typical angina |
| Meeshanthini V. Dogan | 2018 | NA | CHD |
| Zhenzhen Du | 2020 | 3-year follow up | CHD(Positive group); Death, stroke, cancer/tumor, renal failure, rheumatic heart disease, pulmonary heart disease, pericardial defect, heart valve disease, congestive heart failure, acute myocardial infarction(Negative group) |
| Darwin Tay | 2015 | NA | Angina pectoris (AP), coronary insufficiency (CI) and myocardial infarction (MI) |
| Arvind Raghu | 2015 | Only those subjects who had recorded blood cholesterol and blood glucose measurements were included | NA |
| Joshua D. Bundy | 2020 | Age range(45-84) | CVD, history of heart failure, history of myocardial infarction, incomplete variable |
| P.Unnikrishnan | 2016 | >49 years | CVD, died during the follow-up period due to a non-cardiovascular aetiology |
| Zeineb Bouzid | 2021 | NA | Ventricular tachycardia or fibrillation |
| Salah Al-Zaiti | 2020 | NA | Ventricular tachycardia or fibrillation |
| Carlo Ricciardi | 2020 | NA | NA |
| Sebastian Okser | 2010 | A complete set of those SNPs | NA |
| Gilles Chatellier | 2000 | NA | Incomplete variable |
| Jionglin Wu | 2010 | 12 months before the index date | Heart failure |
| Reinhard Voss | 2002 | NA | Deaths due to causes other than CHD within 10 year |
| Matthew W. Segar | 2021 | >40 years | Heart failure |
| Jose Roberto Ayala Solares | 2019 | Restricted the patient cohort to those who had at least one systolic blood pressure recorded within 1 year of baseline (at age 50 years); had at least 10 years of registration with their general practice clinic before baseline, and had at least 3 systolic blood pressure readings recorded within this 10-year period | Cardiovascular disease, receiving treatment for high blood pressure or dyslipidemia |
| Ronilda C. Lacson | 2018 | A SBP of 130 mmHg or higher | Diabetes |
| Wenbing Chang | 2019 | NA | Incomplete variable |
| Gihun Joo | 2020 | NA | Diagnosed with AF (I48), CAD (I21-25), HF (I50), hemorrhagic stroke (HS) (I60-62), or ischemic stroke (IS) (I63-69), HME data contained a null or an incorrect value |
| V. Sree Hari Rao | 2020 | NA | NA |
| Amer M. Johri | 2021 | Age ≥ 18 years, referred for clinically indicated angiography for CAD assessment, the absence of clinical contraindication to angiography | Previous carotid endarterectomy, allergy to perflutren, known or suspected cardiac shunt, previous percutaneous coronary intervention (≥ 1 week) or coronary artery bypass graft surgery, prior myocardial infarction, stroke, or transient ischemic attack (≥ 1 week) |
| Matthew Chun | 2021 | NA | History of stroke or transient ischemic attack at baseline |
| Pei-I Zhang | 2020 | age≥20 years with chest pain | NA |
| Daniel Lindholm | 2018 | Age range (49-69) | Heart failure |
| Konstantia Zarkogianni | 2017 | NA | NA |
| Alexandra K. Lee | 2019 | Included SPRINT participants with CKD, as defined by eGFR (by CKD-Epi creatinine-cystatin C equation) <60 mL/minute per 1.73 m 2 | Participants with missing or invalid biomarker measurements and with missing covariates |
| Ghalib A. Bello | 2015 | NA | NA |
| Anietie U Andy | 2021 | Included patients from inpatient and outpatient settings across two urban academic medical centers, age range(40-79) | ASCVD documented in their EMR |
| Kalliopi Dalakleidi | 2017 | T2DM patients | Experiencing CVD incidence before the first visit |
| Sang-Yeong Cho | 2021 | NA | History of HF, MI, ischemic stroke, prior statin medication, angina with coronary intervention or bypass surgery, statin prescription during the study duration |
| Andrew Lin | 2021 | Age 45–80 years and intermediate risk of CAD based on age (> 55 years in men, > 65 years in women) or the presence of at least one CAD risk factor in younger individuals (age 45–54 years in men or 55–64 years in women) | History of cardiac or cerebrovascular disease or chest pain, prior CAC scanning or invasive coronary angiography, or significant medical co-morbidity |
| Christian Tesche | 2021 | NA | Diagnosed with acute coronary syndrome during the time of care involving the cCTA scan, underwent coronary revascularization within 6 weeks of the cCTA scan, or had a history of previous MI, PCI, or CABG, cCTA data with non-diagnostic image quality was excluded. |
| P.Priyanga | 2020 | NA | NA |
| Aniruddha Dutta Conceptualization | 2020 | NA | NA |
| Premanand Tiwari | 2020 | NA | NA |
| Yunxing Jiang | 2021 | NA | Subjects with a previous history of CVD before the baseline survey |
| Ankush Jamthikar | 2020 | NA | NA |
| Che Ngufor | 2020 | Patients in the FHS data were included in the analysis if they were aged at least 45 years on January 1, 2010 (index date), and were alive on December 31, 2015 | NA |
| Yaqi Zhang | 2021 | NA | Died during the study period or were diagnosed with cardiac dysrhythmia before October 1, 2016（baseline） |
| Dongzhe Hong | 2021 | Patients who had two outpatient diagnoses of T2DM recorded on separate days or a diagnosis recorded during an inpatient encounter | Patients with any missing value in baseline data |
| Kinuka Ogata | 2013 | NA | Were lost to follow-up; incomplete variable |
| Benjamin A. Goldstein | 2014 | All individuals receiving HD were eligible for study inclusion, and there were no exclusions for history of SCA(sudden cardiac arrest ) or presence of an implant device | NA |
| Paolo Emilio Puddu | 2012 | NA | NA |
| Julian Betancur | 2018 | Consecutive patients who were referred for clinically indicated exercise or pharmacological stress MPI | Excluding 70 patients with early revascularization within 90 days |
| Manish Motwani | 2016 | NA | Individuals with known CAD (defined as prior myocardial infarction (MI) or revascularization) or those with early revascularization after the index CCTA (defined as within 90 days) |
| Jacob P. VanHouten | 2014 | The patient be at least 18 years of age at the time of presentation and that they have a troponin measurement and ECG recorded during their visit to the emergency department | NA |
| Fátima Sánchez-Cabo | 2020 | NA | Filtering participants with no ASCVD risk score (n=467, missing blood test data, total cholesterol <130 or >320 mg/dl, high-density lipoprotein (HDL) <20 or >100 mg/dl, systolic blood pressure <90 or >200 mm Hg, statin treatment, or LDL >190 mg/dl), no SCORE (Systematic Coronary Risk Evaluation) risk score (n= 74, reported diabetic), no PESA score (n=74, 2DVUS or CACS data missing), or with missing data in any of the predictors used to build the ML model (n=54 ),  those individuals with either one of these conditions: Total Cholesterol<130 or >320, HDL<20 or >100, SBP<90 or SBP>200, statins treatment or LDL>190. 48 individuals with reported diabetes were further excluded as in the PESA ML subcohort and another 19 were excluded due to incomplete information on the predictors |
| Yafei Wu | 2020 | Age elder than 60 years old in the baseline | Participants with stroke who were missing values for the stroke variable and the predictive variable were excluded from the baseline; Participants with missing values for the outcome variable in 2014 were excluded |
| Georgios Christopoulos | 2020 | They had at least one electrocardiography demonstrating SR within 2 years preceding the baseline MCSA visit | Excluded if they had a history of AF at the time of the baseline visit (or if prior AF status was unknown) |
| Renate M. Hoogeveen | 2020 | NA | NA |
| Agni Orfanoudaki | 2019 | Be stroke-free and above 40 years of age at each baseline examination; the participant was not censored within 10 years from the time of the clinical examination; a stroke-free population at baseline who satisfied the inclusion criteria without censoring | The participant had not experienced a stroke event prior to the date of the baseline clinical examination; exclude younger patients following the paradigm of the R-FSRS model |
| Laila R Bekhet | 2018 | At least three heart failure related encounters had to occur within 12 months; >=50 years old at the time of the first HF diagnose; controls are required to have their first visit within one year of the first office visit of the matching case and have at least one visit a month before or any time after the diagnosis date of the matching case | Exclude all cases that showed any prior history of heart failure, as well as controls with any heart failure incidence before or after the index date with 180 days |
| Christoph Nowak | 2018 | With available outcome data and plasma samples were included in the present investigation, at least mild internal carotid artery stenosis, claudication symptoms with an ankle–brachial pressure in_x005F_x0002_dex ≤0.90, or claudication symptoms with signs of arterial occlusive disease in the ipsilateral extremity on ultrasound ex_x005F_x0002_amination | Proteins with >15% missing values were excluded |
| Alexandros C. Dimopoulos | 2018 | NA | Missing information regarding the development of a combined (fatal or nonfatal) CVD event |
| Juan Zhao | 2019 | Meet the definitions of medical home, each individual to have >=1 visit and >=1 blood pressure measurement during the observation window | Individuals with any CVD diagnosis (ICD-9-CM 411. * or 433. *) prior to the baseline date (i.e. 01/01/2007) were excluded, excluded inpatient physical or laboratory measures for all individuals |
| Shinya Suzuki | 2019 | NA | NA |
| Sabrina Mezzatesta | 2019 | NA | Incomplete variable |
| Ji Min Sung | 2019 | Only those with more than two screenings from 2002–2006 were included | Except those with pre-existing histories of CVD; those who had treatment records of CVD or death, or a history of stroke or heart disease at the baseline were removed |
| Jose A. Quesada | 2019 | NA | NA |
| Randall W. Grout | 2021 | Patients≥18 years, later restricted to age≥40 years, with at least two encounters | Patients were excluded if they had an AF diagnosis prior to 1/1/2016 （outcome period）or had an unknown status of AF in the outcome period |
| Shelda Sajeev | 2021 | NA | Excluded 326 people with a previous CVD history, 6 with missing CVD history data, and 70 with missing CVD outcome data;incomplete variable ,938 with a previous CVD history, 142 with missing CVD history data, and 17 with missing CVD outcome data were excluded; 7035 participants with a previous CVD history and 1867 with missing CVD outcome were excluded |
| David de Gonzalo-Calvo | 2020 | NA | All patients with missing blood samples (n=8, the full case-control pair was discarded even when there was blood sample from one member of the pair: n=16), patients whose plasma samples were used for other experimental procedures (n=10) and patients in which miRNA quantification did not pass the quality control (n=5) were excluded |
| In-Soo Kim | 2020 | age >18 years | a diagnosis of AF before undergoing a health examination, val_x005F_x0002_vular AF, such as mitral valve stenosis or prosthetic valve disease, change of residence to another ZIP code in 2009–2013, missing data on residential ZIP code or in questionnaires for smoking status and alcohol intake completed during the health examinations |
| Michael SCHREMPF | 2021 | age >=18 years | Patients with the following ICD 10 codes were excluded: intracerebral haemorrhage (I61, I62), aneurysm and dissection (I72) |
| Simon Nusinovici | 2020 | NA | Ineligible participants included people who died, who migrated and who were prisoners between baseline and follow-up visit |
| Luca Navarini | 2020 | NA | NA |
| Divneet Mandair | 2020 | NA | Extracting patients that had MI, excluding patients who had a prior diagnosis of MI |

**Table S3. The definition and measurement of outcomes**

| **Outcomes** | **Definition** | **Measurement methods** |
| --- | --- | --- |
| CVD mortality | Cardiovascular mortality: Death from a clearly identifiable CV cause or death of subjects with a history of CV disease and no identifiable non-CV cause for death**[31]** | Social Security Death Index and California Death Registry Veterans Affairs medical records |
|  | Cardiac death: Death due to cardiovascular etiology (ICD-10 codes, Ixx)**[72]** | NA |
|  | CVD mortality: Deaths that occurred within 15 years of baseline, with CVD listed as the primary or secondary cause of death based on International Classification of Diseases (ICD) from the 9th (390–459) and 10th (I00–I99) revisions**[103]** | NA |
|  | CVD death: death from CHD, stroke, congestive heart failure, and other circulatory disease**[34]** | Social Security Death Index Death Master File |
|  | Death from CVD myocardial infarction, coronary arterial intervention or bypass surgery stroke: ICD-10 codes I-00 to I-99**[37]** | NA |
|  | Sudden cardiac arrest death: NA**[84]** | NA |
| All-cause mortality | All-cause mortality: NA**[87]** | In U.S. sites, death status was ascertained by querying the Social Security Death Index.In non-U.S. sites, follow-up data were collected by mail or telephone contact with the patients or their families; events were verified by hospital records or contacts with the attending physician |
|  | All-cause mortality: NA**[38]** | NA |
|  | All-cause mortality: NA**[65]** | NA |
|  | All-cause mortality: NA**[98]** | NA |
|  | All-cause mortality: Death records in the REP and FHS were obtained from the Minnesota Electronic Death Certificates and the National Death Index(ICD)**[80]** | Death records in the REP and FHS were obtained from the Minnesota Electronic Death Certificates and the National Death Index(ICD) |
|  | All-cause mortality: NA**[69]** | NA |
|  | All-cause mortality: NA**[85]** | NA |
|  | All-cause mortality: NA**[32]** | Statistics Korea |
| CHD death | Atherosclerotic death: NA**[83]** | A review of obituaries, medical records, death certificates, hospital charts, and interviews with primary care physicians, families of the deceased and other witnesses |
|  | CHD death: NA**[34]** | Social Security Death Index Master File |
| Fatal or nonfatal CVD(complete CVD) | CVD: Hard CVD+congestive heart failure, transient ischemic attack, peripheral vascular disease, resuscitated cardiac arrest, percutaneous transluminal coronary angioplasties, probable angina, other revascularization, other CVD death, other atherosclerotic death, and cardiac bypass graft surgery**[35]** | NA |
|  | CVD: hypertension, hyperlipidemia, myocardial infarction, and angina pectoris**[36]** | NA |
|  | CVD: Coronary heart disease, ischaemic stroke, or transient ischaemic attack**[40]** | Same as for QRISK3 |
|  | CVD: AP, CI, MI, transient ischemic attack (TIA), stroke and congestive heart failure (CHF)**[46]** | Surveillance of hospital discharges, subsequent examinations, death certificates and autopsy records |
|  | CVD: NA**[47]** | NA |
|  | CVD: myocardial infarction, stroke, bypass surgery for coronary artery disease(CAD), or death from CAD, self-reported angina**[49]** | NA |
|  | CVD: NA**[75]** | NA |
|  | CVD: Coronary heart disease【Discharge diagnosis (ICD-10 codes, I20 –I25) with coronary revascularization】+ Cerebrovascular events 【Discharge diagnosis (ICD-10 codes, I60–I64 and S64–S66) with brain imaging】+ Peripheral artery disease【Discharge diagnosis (ICD-10 codes, I708, I709, I743, I744, I745, I771)】+ Heart failure【Discharge diagnosis (ICD-10 code, I50)】**[72]** | NA |
|  | CVD: Cardiovascular mortality【Death due to cardiovascular etiology (ICD-10 codes, I20-I25, I63, I64, and G45)】+ Coronary heart disease【Discharge diagnosis (ICD-10 codes, I20 –I25) with coronary revascularization】+ Stroke【Discharge diagnosis (ICD-10 codes, I61) with brain imaging】+ Transient ischemic accident【Diagnosis (ICD-10 code, G45) with brain imaging】**[72]** | NA |
|  | CVD: A CVD event was defined as hospitalization or death during follow-up period for ischemic heart disease, cerebrovascular disease, or other related diseases (ICD9: Codes 390–495)**[78]** | Identified CVD events from local hospital medical records, health insurance claims, questionnaire responses, death registries from the morbidity and mortality surveillance system, and questionnaire responses during follow-up period. |
|  | CVD/stroke: NA**[79]** | NA |
|  | CVD: (1) death from CVD (International Classification of Diseases 10th edition [ICD-10] codes) (2) hospitalization due to myocardial infarction, coronary arterial intervention or bypass surgery (3) hospitalization due to stroke**[100]** | NA |
|  | CVD: Individuals with ≥1 CVD diagnosis codes (the International Classifcation of Diseases, Ninth Revision, Clinical Modifcation [ICD-9-CM]: 411. * and 433. *) within the 10-year prediction window**[97]** | NA |
|  | CVD: NA**[99]** | NA |
|  | CVD: Self-reported using an interview-based, standardized questionnaire, defined as a history of myocardial infarction, angina pectoris or stroke**[107]** | NA |
|  | CVD: Myocardial infarction or unstable angina pectoris, stable angina pectoris, transient ischaemic attack, peripheral artery disease and heart failure**[108]** | NA |
|  | CVD: AF, CAD, HF, HS, and IS**[61]** | NA |
|  | CVD: First hospitalization for, or death from, coronary heart disease or stroke (including transient ischemic attack), identified from 3 different sources (general practice records, hospitalization, and mortality databases) using the ICD-10, and relevant UK Read codes**[58]** | NA |
|  | CVD: A composite of CVD death, stroke, and CHD**[38]** | NA |
|  | CVD: a composite of four events (HF events, ACS events, IS events, and intracranial hemorrhage events)**[98]** | Each event comprising a cardiovascular event was determined when it required hospitalization |
|  | CVD: All-cause mortality or hospital admission due to stroke (International Classification of Diseases 9 [ICD - 9] codes 430 –438, 444) or ischemic cardiopathy (ICD -9 410 -414)**[101]** | Hospitalizations and CVD mortality were assessed by reviewing hospital records and annual mortality data during follow -up |
|  | CVD: Defined as a combined endpoint of coronary heart disease (myocardial infarction, unstable angina, coronary revascularization, silent ischaemia) and cerebrovascular disease (ischaemic stroke and transient ischaemic attack)  1 either hospitalization or death with ICD code I2122 coded as the underlying cause 2 The presence of documented stenosis or vascular damage on aorta and limb arteries**[92]** | NA |
|  | CVD: Coronary revascularization (i.e. percutaneous coronary intervention, coronary artery bypass surgery), heart failure, nonfatal myocardial infarction, stroke, or cardiac death**[63]** | NA |
|  | Current atherosclerotic cardiovascular disease: NA**[70]** | NA |
|  | Hard ASCVD: Cardiovascular mortality[Death due to cardiovascular etiology (ICD-10 codes, I20-I25)] + Coronary heart disease【Discharge diagnosis (ICD-10 codes, I20 –I25) with coronary revascularization】+ Cerebrovascular events【Discharge diagnosis (ICD-10 codes, I60–I64 and S64–S66) with brain imaging】**[72]** | NA |
|  | ASCVD: First acute myocardial infarction,stroke, or fatal coronary artery disease(ICD-9 and ICD-10)**[33]** | NA |
|  | Hard CVD: Myocardial infarction, fatal CHD,stroke, and stroke death**[35]** | NA |
|  | Hard adverse cardiovascular events: MI or/and cardiac death**[42]** | National Death Index query and by comprehensive review of corresponding medical, hospital, and death records |
|  | Fatal or non-fatal CVD event: ICD-10 vascular dementia; coronary/ischaemic heart diseases; heart failure events, including acute and chronic systolic heart failures; cerebrovascular diseases; ischemic heart disease; cerebrovascular disease**[39]** | NA |
|  | Fatal or non-fatal CVD incidence: Coronary heart disease, angina, heart failure or stroke, according to ICD-9 classification**[96]** | NA |
|  | Fatal or non-fatal CVD incidence: Stroke and CHD**[67]** | NA |
|  | Fatal or nonfatal CVD: NA**[71]** | NA |
|  | Fatal or non-fatal cardiovascular event: ICD-10 codes I20 to I25 coronary (ischaemic) heart conditions; I60 to I69 cerebrovascular conditions.**[41]** | Primary or secondary care computerised record |
|  | Major adverse cardiovascular events (MACE): Myocardial infarction (MI), late revascularization (occurring > 180 days after the CT), or cardiac death**[73]** | NA |
|  | Major adverse cardiovascular events (MACE): Cardiac death (fatal myocardial infarction [MI]), non-fatal MI (ST segment elevation [STEMI] and non-ST segment elevation MI [NSTEMI]), and unstable angina leading to coronary revascularization (percutaneous coronary intervention [PCI] or coronary artery bypass grafting [CABG]) with more than 6 weeks between cCTA and invasive coronary angiography (ICA) with the revascularization procedure**[74]** | NA |
|  | Major adverse cardiovascular events (MACE): We defined MCE as a composite of myocardial infarction, stroke, percutaneous transluminal coronary angioplasty, use of cardiac devices, coronary artery procedures, congestive heart failure, ischemic heart disease, coronary artery disease, cardiomyopathy, cardiac arrest, or angina occurring in the follow-up period(ICD)**[80]** | NA |
|  | Major adverse cardiovascular events (MACE): Consisted of all-cause mortality, nonfatal myocardial infarction, unstable angina, or late coronary revascularization (percutaneous coronary intervention or coronary artery bypass grafting)**[86]** | All-cause mortality was determined from the Social Security Death Index and combined with MACE obtained from the hospital electronic medical records, including all clinics, as well as cardiology group and hospital visits. Nonfatal myocardial infarction was defined based on the criteria of hospital admission for chest pain, elevated cardiac enzyme levels, and typical changes on the electrocardiogram |
|  | Major adverse cardiovascular events (MACE): A new episode of fatal or non-fatal myocardial infarc_x005F_x0002_tion (I21 in ICD-10; www.who.int/classifications/icd/en/) or fatal/non-fatal stroke (I60–I63), whichever occurred first, and was from obtained from hospital and death register linkage**[95]** | NA |
|  | Major adverse cardiovascular events (MACE): Death from cardiovascular causes, non-fatal myocardial infarction or non-fatal stroke. A sudden, unexpected death was attributed to CHD, definite or suspected, if there was inadequate information to ascribe a non_x005F_x0002_cardiovascular cause**[104]** | NA |
|  | Major adverse cardiovascular events (MACE): All patients with a coded diagnosis of angina pectoris (ICD-10 code I20) with a manual description associated to an acute coronary syndrome**[106]** | Transfer data, diagnoses and procedures were used |
| Stroke | Stroke: ICD-10**[64]** | Trained medical staff using the International Classification of Diseases 10th revision (ICD-10) |
|  | Stroke: An acute onset focal neurological deficit of vascular etiology, persisting for more than 24 hours, concordant with the World Health Organization (WHO) definition; both ischemic and hemorrhagic strokes were included as in the original FSRS and updated R-FSRS**[93]** | NA |
|  | Stroke: Are you suffering from stroke (yes/no)?**[90]** | The elderly patients or their family members self-reporte the answer of the question |
|  | Stroke: ICD-9 CM codes: 430-436, ICD10 CM codes: I60-I66**[82]** | NA |
|  | Stroke: Stroke was defined as rapid onset of a documented focal neurologic deficit (vascular causes) lasting 24 hours or until death, or if < 24 hours, when there was a clinically relevant brain lesion**[38]** | NA |
|  | Ischemic stroke: NA**[98]** | Each event comprising a cardiovascular event was determined when it required hospitalization |
| CHD | CHD: NA**[44]** | A panel of three investigators on the Framingham Endpoint Review Committee |
|  | CHD: Coronary heart disease, coronary sclerosis heart disease, ischemic cardiomyopathy, angina, acute myocardial infarction, myocardial ischemia, heart failure (all translated from Chinese), and others,(ICD)-10 [40] diagnostic codes I20 to I25 or the keywords related to CHD conditions**[45]** | NA |
|  | CHD: Stenosis equal or above 70% were labelled as diseased**[43]** | Surgical Coronary Angiography final medical report |
|  | CHD: Presence of sudden cardiac death or of a definite fatal or non-fatal myocardial infarction on the basis of ECG and/or cardiac enzyme changes**[56]** | ECG and/or cardiac enzyme changes |
|  | CHD: CHD:≥ 50% stenosis**[63]** | Angiography and scored by expert researchers |
|  | CHD: Yes response to the question "Have you been ever told you had coronary heart disease?"**[76]** | NA |
|  | CHD: ICD-9 CM codes: 410-414,429.2; ICD-10 CM codes: I20-I25**[82]** | NA |
|  | CHD: Criteria for CHD included any of myocardial infarction, resuscitated cardiac arrest, definite angina, probable angina followed by revascularization and CHD death**[38]** | NA |
|  | CHD: NA**[62]** | NA |
| AF | AF: Each diagnosis, based on the 10th revision of the International Clas_x005F_x0002_sifcation of Disease codes, was defned as the frst occurrence during at least two diferent days of outpatient hospital visits or on the frst hospital admission**[105]** | AF was diagnosed on the basis of hospital admission or at least two outpatient visits for AF |
|  | AF: An International Statistical Classifcation of Diseases and Related Health Problems (ICD) 10th Edition diagnosis of I48.0, I48.1, I48.2, I48.3, I48.4, I48.91, I48.92, or ICD 9th Edition diagnosis of 427.31, 427.32, or a mention within an ECG report of "atrial fibrillation", "atrial futter", "Afb", "AFl", "A-fb" or "A-f" using text matching**[102]** | NA |
|  | AF: ICD-9 diagnosis codes for AF (427.31) or atrial flutter (427.32)**[48]** | study electrocardiograms and hospital discharge diagnosis ICD-9 codes |
|  | AF: NA**[77]** | NA |
|  | AF: NA**[91]** | NA |
|  | AF: Criteria for incident AF as an end-point required in-hospital AF diagnosis according to ICD9 codes**[38]** | NA |
| Heart failure | Heart failure: (1) heart failure diagnosis appeared on the problem list at least once; (2) heart failure appeared in the EHR for 2 outpatient encounters,indicating consistency in clinical assessment; (3) at least 2 medications were prescribed with an associated ICD-9 diag- nosis of heart failure; or (4) heart failure appeared on 1 or more outpatient encounter and at least 1 medication was prescribed with an associated ICD-9 diagnosis for heart failure**[55]** | NA |
|  | Heart failure: ICD-10:I50**[66]** | Hospital Episode Statistics register ICD-10:I50 |
|  | Heart failure: ICD-9 CM codes: 402.01,402.11,402.91,428, ICD-10 code: I50**[82]** | NA |
|  | Heart failure: ICD-9 and ICD-10**[94]** | NA |
|  | Heart failure: NA**[98]** | Each event comprising a cardiovascular event was determined when it required hospitalization |
|  | Heart failure: symptomatic HF diagnosed by a physician for a patient receiving medical treatment for HF and 1) pulmonary edema/congestion, and/or 2) dilated ventricle or poor LV function, or evidence of LV diastolic dysfunction**[38]** | NA |
|  | Heart failure: First hospitalization event with HF in each of the study cohorts**[57]** | NA |
|  | Cardio heart failure: NA**[99]** | NA |
|  | Chronic heart failure: NA**[52]** | NA |
| Acute coronary syndrome | Acute coronary syndrome: Myocardial infarction（4th Universal Definition of MI guidelines） or unstable angina**[51]** | NA |
|  | Acute coronary syndrome: An umbrella term for unstable angina and myocardial infarction（ICD-9)**[88]** | A patient with ACS as one who visited the emergency department for chest pain and who received any ICD-9 code listed as an ACS diagnostic code, or any ICD-9 or CPT procedure code for percutaneous coronary intervention (PCI) or coronary artery bypass graft (CABG) over the thirty days following the admission date. If a patient was admitted twice within a short amount of time, and within thirty days of both visits received a code consistent with ACS, both admission instances were considered positive for an ACS event. |
|  | Acute coronary syndrome: Myocardial infarction （4th Universal Definition of MI guidelines）or unstable angina**[50]** | NA |
|  | Acute coronary syndrome: NA**[98]** | Each event comprising a cardiovascular event was determined when it required hospitalization |
| Myocardial infarction (MI) | Myocardial infarction (MI): ICD-9**[109]** | NA |
|  | Acute myocardial infarction (AMI): (ICD-9-CM: 410–414 or ICD-10: I20-I25) within 1 month**[65]** | NA |
| Arrythmia | Arrythmia: NA**[99]** | NA |
|  | Cardiac dysrhythmia: Cardiac dysrhythmia was defined according to ICD-10-CM, including paroxysmal supraventricular tachycardia, paroxysmal ventricular tachycardia, atrial fibrillation, atrial flutter, premature beats, sinoatrial node dysfunction, and other cardiac dysrhythmias (ICD-10-CM diagnosis codes from I47 to I49)**[81]** | NA |
| Increased Pre-Clinical Carotid Atherosclerosis | NA**[53]** | NA |
| Subclinical atherosclerosis | the presence of ≥1 atherosclerotic plaque in the peripheral territories orCACS ≥0.5**[89]** | the extent of SA was defined as the presence of plaque or CACS >=0.5 for each vascular site (right and left carotid arteries, aorta, right and left femoral arteries, and coronary arteries) |
| Myocardial infarction,stroke or cardiovascular death | NA**[54]** | NA |
| Hypertensive heart disease | NA**[60]** | NA |
| CVD events, heart failure (HF), all-cause mortality | CVD events, defined as a composite of myocardial infarction, acute coronary syndrome, stroke, acute decompensated HF, and cardiovascular death; all-cause mortality and HF, defined as hospitalization or emergency department visit with signs or symptoms of HF and requiring infusion therapy treatment.**[68]** | All-cause mortality and HF, defined as hospitalization or emergency department visit with signs or symptoms of HF and requiring infusion therapy treatment |
| Intracranial hemorrhage | NA**[98]** | Each event comprising a cardiovascular event was determined when it required hospitalization |
| Ischemia heart disease | NA**[99]** | NA |
| Cardio-cerebroVascular diseases | NA**[99]** | NA |
| Myocardial infarction, other acute coronary syndromes, stroke, heart failure or death from a cardiovascular event | NA**[59]** | NA |

**Table S4. The counting and characteristics of algorithms**

| **Categories** | **Idiographic algorithms** | **N**umber |
| --- | --- | --- |
| **LogR** |  |  |
|  | LogR | 74 |
| **RF** |  |  |
|  | RF | 58 |
|  | RSF | 13 |
| **NN** |  |  |
|  | ANN | 4 |
|  | EANN | 1 |
|  | BPN | 1 |
|  | FNN | 3 |
|  | CNN | 13 |
|  | SNN | 4 |
|  | PNN | 1 |
|  | HWNNs | 2 |
|  | MLP | 5 |
|  | SOMs | 1 |
|  | DNN | 5 |
|  | ANCSc | 1 |
|  | LSTM | 6 |
|  | RETAIN | 1 |
|  | DBN | 1 |
|  | Neural network | 14 |
| **SVM** |  |  |
|  | SVM | 21 |
|  | LSVM | 3 |
|  | NLSVM (radial) | 1 |
|  | SVC | 2 |
|  | SVCR | 10 |
|  | SVCP | 10 |
|  | SVCL | 10 |
| **Boosting** |  |  |
|  | AdaBoost | 13 |
|  | logitboost | 5 |
|  | XGBoost | 14 |
|  | GBDT/MART/GBT/GBM | 20 |
|  | LightGBM | 2 |
|  | Generalized boosted regression | 2 |
| **Cox** |  |  |
|  | Cox | 51 |
| **Bayesian** |  |  |
|  | Bayesian analysis | 2 |
|  | Bayesian non-linear | 1 |
|  | naïve Bayes | 21 |
|  | Gaussian naive Bayes | 1 |
|  | Bayesian Lasso | 2 |
| **DT** |  |  |
|  | CART | 15 |
|  | RPART | 1 |
|  | OCT | 2 |
|  | DT | 5 |
| **KNN** |  |  |
|  | KNN | 22 |
| **LinR** |  |  |
|  | GLM(EN) | 4 |
|  | LDA | 4 |
| **Combination** |  |  |
|  | RF+SVM | 1 |
|  | XGBoost+SVM | 1 |
|  | GBDT+SVM | 1 |
|  | Proposed RNN-LCBWO | 1 |
|  | LR+GBM+ANN | 2 |
| **Others** |  |  |
|  | PSM | 3 |
|  | PSO | 2 |
|  | R-FSRS | 2 |
|  | SSPR | 2 |
|  | Bagging | 1 |
|  | EDC-AIRS | 1 |
|  | ET | 1 |
|  | FCM | 1 |
|  | FL | 1 |
|  | QDA | 1 |
|  | RUSBoost | 1 |
| **Automated-ML** |  |  |
|  | autoML (h2o) | 1 |
|  | AutoPrognosis | 1 |
|  | Auto ECG read | 2 |
|  | Expert ECG read | 2 |
|  | Automated ML (DataRobot) | 6 |
|  |  |  |

LogR: Logistic Regression; RF: Random Forest; RSF: Random Survival Forest; NN: Neural Networks; ANN: Artificial Neural Networks; EANN: Ensembles of Artificial Neural Networks; BPN: Back Propagation Networks; FNN: Feedforward Neural Networks; CNN: Convolutional Neural Networks; SNN: Shallow Neural Networks; PNN: Probabilistic Neural Networks; HWNNs: Hybrid Wavelet Neural Networks; MLP: MultiLayer Perceptron models; SOMs: Self Organizing Maps; DNN: Deep Neural Networks; ANCSc: Artificial Neural Cell System For Classification; LSTM: Long Short Term Memory Network; RETAIN: The Reverse Time Attention Model; DBN: Deep Belief Networks; SVM: Support Vector Machine; LSVM: LinearSVM; NLSVM: NonLinearSVM; SVC: Support Vector Classifier; SVCR: Radial SVC; SVCP: SVC with Polynomial kernel; SVCL: LinearSVC; AdaBoost: Adaptive Boosting; XGBoost: Extreme Gradient Boosting; GBDT: Gradient Boosting Decision Tree-Gradient Boosting Classifier; MART: Multiple Additive Regression Tree; GBT: Gradient Boosted Tree; GBM: Gradient Boosting Machine; Cox: Cox regression model; CART: Classification And Regression Tree ; RPART: Recursive Partitioning and Regression Trees; OCT: Optimal Classification Trees; DT: Decision Tree; KNN: K-Nearest Neighbor; GLM(EN): Generalized Linear Model with Elastic Net Regularization; LDA: Linear Discriminant Analysis; Proposed RNN-LCBWO: Hybrid Recurrent Neural Network-Logistic Chaos-Based Whale Optimization Structured Hybrid Framework; PSM: Parametric Survival Model; PSO: Particle Swarm Optimization; R-FSRS: Revised Free Space Repetition Scheduler; SSPR: Supervised Statistical Pattern Recognition; EDC-AIRS: Evolutionary Data-Conscious Artificial Immune Recognition System; ET: Extra Trees; FCM: Fuzzy Cognitive Maps; FL: Fuzzy Logic; QDA: Quadratic Discriminant Analysis; RUSBoost: Random Under Sampling Boosting.

**Table S5. Risk of bias assessment of prediction models**

| **First author** | **Models** | **Publication year** | **Participants** | **Predictors** | **Outcomes** | **Analysis** | **Overall** |
| --- | --- | --- | --- | --- | --- | --- | --- |
| Marco V. Perez[31] | 1 | 2009 | High | Unclear | Low | High | High |
| Donghee Han[32] | 1 | 2019 | High | Unclear | High | High | High |
| Donghee Han[32] | 2 | 2019 | High | Unclear | High | High | High |
| Donghee Han[32] | 3 | 2019 | Unclear | Unclear | High | High | High |
| Donghee Han[32] | 4 | 2019 | Unclear | Unclear | High | High | High |
| Andrew Ward[33] | 1 | 2020 | High | Unclear | Unclear | High | High |
| Andrew Ward[33] | 2 | 2020 | High | Unclear | Unclear | High | High |
| Andrew Ward[33] | 3 | 2020 | High | Unclear | Unclear | High | High |
| Andrew Ward[33] | 4 | 2020 | High | Unclear | Unclear | High | High |
| Andrew Ward[33] | 5 | 2020 | High | Unclear | Unclear | High | High |
| Andrew Ward[33] | 6 | 2020 | High | Unclear | Unclear | High | High |
| Andrew Ward[33] | 7 | 2020 | High | Unclear | Unclear | High | High |
| Andrew Ward[33] | 8 | 2020 | High | Unclear | Unclear | High | High |
| Rine Nakanishi[34] | 1 | 2020 | Unclear | Unclear | High | High | High |
| Rine Nakanishi[34] | 2 | 2020 | Unclear | Unclear | High | High | High |
| Ioannis A. Kakadiaris[35] | 1 | 2018 | Low | Unclear | Unclear | High | High |
| Ioannis A. Kakadiaris[35] | 2 | 2018 | Low | Unclear | Unclear | High | High |
| Ioannis A. Kakadiaris[5][35] | 3 | 2018 | Low | Unclear | Unclear | High | High |
| Ioannis A. Kakadiaris[35] | 4 | 2018 | Low | Unclear | Unclear | High | High |
| Jaekwon Kim[36] | 1 | 2017 | Low | Unclear | Unclear | High | High |
| Jaekwon Kim[36] | 2 | 2017 | Low | Unclear | Unclear | High | High |
| Jaekwon Kim[36] | 3 | 2017 | Low | Unclear | Unclear | High | High |
| Jaekwon Kim[36] | 4 | 2017 | Low | Unclear | Unclear | High | High |
| Jaekwon Kim[36] | 5 | 2017 | Low | Unclear | Unclear | High | High |
| Jaekwon Kim[36] | 6 | 2017 | Low | Unclear | Unclear | High | High |
| In-Jeong Cho[37] | 1 | 2020 | Low | Unclear | Unclear | High | High |
| In-Jeong Cho[37] | 2 | 2020 | Low | Unclear | Unclear | High | High |
| In-Jeong Cho[37] | 3 | 2020 | Low | Low | Unclear | High | High |
| Bharath Ambale-Venkatesh[38] | 1 | 2017 | Low | Unclear | Unclear | High | High |
| Bharath Ambale-Venkatesh[38] | 2 | 2017 | Low | Unclear | Unclear | High | High |
| Bharath Ambale-Venkatesh[38] | 3 | 2017 | Low | Unclear | Unclear | High | High |
| Bharath Ambale-Venkatesh[38] | 4 | 2017 | Low | Unclear | Unclear | High | High |
| Bharath Ambale-Venkatesh[38] | 5 | 2017 | Low | Unclear | Unclear | High | High |
| Bharath Ambale-Venkatesh[38] | 6 | 2017 | Low | Unclear | Unclear | High | High |
| Bharath Ambale-Venkatesh[38] | 7 | 2017 | Low | Unclear | Unclear | High | High |
| Bharath Ambale-Venkatesh[38] | 8 | 2017 | Low | Unclear | Unclear | High | High |
| Bharath Ambale-Venkatesh[38] | 9 | 2017 | Low | Unclear | Unclear | High | High |
| Bharath Ambale-Venkatesh[38] | 10 | 2017 | Low | Unclear | Unclear | High | High |
| Bharath Ambale-Venkatesh[38] | 11 | 2017 | Low | Unclear | Unclear | High | High |
| Bharath Ambale-Venkatesh[38] | 12 | 2017 | Low | Unclear | Unclear | High | High |
| Bharath Ambale-Venkatesh[38] | 13 | 2017 | Low | Unclear | Unclear | High | High |
| Bharath Ambale-Venkatesh[38] | 14 | 2017 | Low | Unclear | Unclear | High | High |
| Bharath Ambale-Venkatesh[38] | 15 | 2017 | Low | Unclear | Unclear | High | High |
| Bharath Ambale-Venkatesh[38] | 16 | 2017 | Low | Unclear | Unclear | High | High |
| Bharath Ambale-Venkatesh[38] | 17 | 2017 | Low | Unclear | Unclear | High | High |
| Bharath Ambale-Venkatesh[38] | 18 | 2017 | Low | Unclear | Unclear | High | High |
| Ahmed M. Alaa[39] | 1 | 2019 | Low | Unclear | Unclear | High | High |
| Yan Li[40] | 1 | 2020 | Low | Unclear | Unclear | High | High |
| Yan Li[40] | 2 | 2020 | Low | Unclear | Unclear | High | High |
| Yan Li[40] | 3 | 2020 | Low | Unclear | Unclear | High | High |
| Yan Li[40] | 4 | 2020 | Low | Unclear | Unclear | High | High |
| Yan Li[40] | 5 | 2020 | Low | Unclear | Unclear | High | High |
| Yan Li[40] | 6 | 2020 | Low | Unclear | Unclear | High | High |
| Yan Li[40] | 7 | 2020 | Low | Unclear | Unclear | High | High |
| Yan Li[40] | 8 | 2020 | Low | Unclear | Unclear | High | High |
| Yan Li[40] | 9 | 2020 | Low | Unclear | Unclear | High | High |
| Yan Li[40] | 10 | 2020 | Low | Unclear | Unclear | High | High |
| Yan Li[40] | 11 | 2020 | Low | Unclear | Unclear | High | High |
| Yan Li[40] | 12 | 2020 | Low | Unclear | Unclear | High | High |
| Yan Li[40] | 13 | 2020 | Low | Unclear | Unclear | High | High |
| Yan Li[40] | 14 | 2020 | Low | Unclear | Unclear | High | High |
| Yan Li[40] | 15 | 2020 | Low | Unclear | Unclear | High | High |
| Yan Li[40] | 16 | 2020 | Low | Unclear | Unclear | High | High |
| Stephen F. Weng[41] | 1 | 2017 | Low | Unclear | Unclear | High | High |
| Stephen F. Weng[41] | 2 | 2017 | Low | Unclear | Unclear | High | High |
| Stephen F. Weng[41] | 3 | 2017 | Low | Unclear | Unclear | High | High |
| Stephen F. Weng[41] | 4 | 2017 | Low | Unclear | Unclear | High | High |
| Frederic Commandeur[42] | 1 | 2020 | High | Low | Unclear | High | High |
| Ioannis D. Apostolopoulos[43] | 1 | 2020 | High | Unclear | High | High | High |
| Meeshanthini V. Dogan[44] | 1 | 2018 | Low | Unclear | Unclear | High | High |
| Meeshanthini V. Dogan[44] | 2 | 2018 | Low | Unclear | Unclear | High | High |
| Zhenzhen Du[45] | 1 | 2020 | High | Unclear | Unclear | High | High |
| Zhenzhen Du[45] | 2 | 2020 | High | Unclear | Unclear | High | High |
| Zhenzhen Du[45] | 3 | 2020 | High | Unclear | Unclear | High | High |
| Zhenzhen Du[45] | 4 | 2020 | High | Unclear | Unclear | High | High |
| Zhenzhen Du[45] | 5 | 2020 | High | Unclear | Unclear | High | High |
| Zhenzhen Du[45] | 6 | 2020 | High | Unclear | Unclear | High | High |
| Darwin Tay[46] | 1 | 2015 | Low | Unclear | Unclear | High | High |
| Darwin Tay[46] | 2 | 2015 | Low | Unclear | Unclear | High | High |
| Darwin Tay[46] | 3 | 2015 | Low | Unclear | Unclear | High | High |
| Arvind Raghu[47] | 1 | 2015 | Low | Unclear | Unclear | High | High |
| Arvind Raghu[47] | 2 | 2015 | Low | Unclear | Unclear | High | High |
| Arvind Raghu[47] | 3 | 2015 | Low | Unclear | Unclear | High | High |
| Joshua D. Bundy[48] | 1 | 2020 | Low | Low | Unclear | High | High |
| P.Unnikrishnan[49] | 1 | 2016 | Low | Unclear | Unclear | High | High |
| P.Unnikrishnan[49] | 2 | 2016 | Low | Unclear | Unclear | High | High |
| Zeineb Bouzid[50] | 1 | 2021 | Low | Low | High | High | High |
| Zeineb Bouzid[50] | 2 | 2021 | Low | Low | High | High | High |
| Zeineb Bouzid[50] | 3 | 2021 | Low | Low | High | High | High |
| Zeineb Bouzid[50] | 4 | 2021 | Low | Low | High | High | High |
| Salah Al-Zaiti[51] | 1 | 2020 | Low | Low | High | High | High |
| Salah Al-Zaiti[51] | 2 | 2020 | Low | Low | High | High | High |
| Salah Al-Zaiti[51] | 3 | 2020 | Low | Low | High | High | High |
| Salah Al-Zaiti[51] | 4 | 2020 | Low | Low | High | High | High |
| Salah Al-Zaiti[51] | 5 | 2020 | Low | Low | High | High | High |
| Salah Al-Zaiti[51] | 6 | 2020 | Low | Low | High | High | High |
| Carlo Ricciardi[52] | 1 | 2020 | Low | Low | Unclear | High | High |
| Carlo Ricciardi[52] | 2 | 2020 | Low | Low | Unclear | High | High |
| Carlo Ricciardi[52] | 3 | 2020 | Low | Low | Unclear | High | High |
| Sebastian Okser[53] | 1 | 2010 | Low | Low | Unclear | High | High |
| Gilles Chatellier[54] | 1 | 2000 | Low | Unclear | Unclear | High | High |
| Gilles Chatellier[54] | 2 | 2000 | Low | Unclear | Unclear | High | High |
| Gilles Chatellier[54] | 3 | 2000 | Low | Unclear | Unclear | High | High |
| Jionglin Wu[55] | 1 | 2010 | Low | Unclear | Unclear | High | High |
| Jionglin Wu[55] | 2 | 2010 | Low | Unclear | Unclear | High | High |
| Jionglin Wu[55] | 3 | 2010 | Low | Unclear | Unclear | High | High |
| Reinhard Voss[56] | 1 | 2002 | Low | Unclear | Unclear | High | High |
| Reinhard Voss[56] | 2 | 2002 | Low | Unclear | Unclear | High | High |
| Reinhard Voss[56] | 3 | 2002 | Low | Unclear | Unclear | High | High |
| Matthew W. Segar[57] | 1 | 2021 | High | Unclear | Unclear | High | High |
| Matthew W. Segar[57] | 2 | 2021 | High | Unclear | Unclear | High | High |
| Matthew W. Segar[57] | 3 | 2021 | High | Unclear | Unclear | High | High |
| Matthew W. Segar[57] | 4 | 2021 | High | Unclear | Unclear | High | High |
| Matthew W. Segar[57] | 5 | 2021 | High | Unclear | Unclear | High | High |
| Matthew W. Segar[57] | 6 | 2021 | High | Unclear | Unclear | High | High |
| Matthew W. Segar[57] | 7 | 2021 | High | Unclear | Unclear | High | High |
| Matthew W. Segar[57] | 8 | 2021 | High | Unclear | Unclear | High | High |
| Matthew W. Segar[57] | 9 | 2021 | High | Unclear | Unclear | High | High |
| Matthew W. Segar[57] | 10 | 2021 | High | Unclear | Unclear | High | High |
| Matthew W. Segar[57] | 11 | 2021 | High | Unclear | Unclear | High | High |
| Matthew W. Segar[57] | 12 | 2021 | High | Unclear | Unclear | High | High |
| Matthew W. Segar[57] | 13 | 2021 | High | Unclear | Unclear | High | High |
| Matthew W. Segar[57] | 14 | 2021 | High | Unclear | Unclear | High | High |
| Matthew W. Segar[57] | 15 | 2021 | High | Unclear | Unclear | High | High |
| Matthew W. Segar[57] | 16 | 2021 | High | Unclear | Unclear | High | High |
| Matthew W. Segar[57] | 17 | 2021 | High | Unclear | Unclear | High | High |
| Matthew W. Segar[57] | 18 | 2021 | High | Unclear | Unclear | High | High |
| Matthew W. Segar[57] | 19 | 2021 | High | Unclear | Unclear | High | High |
| Matthew W. Segar[57] | 20 | 2021 | High | Unclear | Unclear | High | High |
| Matthew W. Segar[57] | 21 | 2021 | High | Unclear | Unclear | High | High |
| Matthew W. Segar[57] | 22 | 2021 | High | Unclear | Unclear | High | High |
| Matthew W. Segar[57] | 23 | 2021 | High | Unclear | Unclear | High | High |
| Matthew W. Segar[57] | 24 | 2021 | High | Unclear | Unclear | High | High |
| Jose Roberto Ayala Solares[58] | 1 | 2019 | Low | Low | Unclear | High | High |
| Ronilda C. Lacson[59] | 1 | 2018 | High | Unclear | Unclear | High | High |
| Wenbing Chang[60] | 1 | 2019 | Unclear | Unclear | High | High | High |
| Wenbing Chang[60] | 2 | 2019 | Unclear | Unclear | High | High | High |
| Wenbing Chang[60] | 3 | 2019 | Unclear | Unclear | High | High | High |
| Wenbing Chang[60] | 4 | 2019 | Unclear | Unclear | High | High | High |
| Wenbing Chang[60] | 5 | 2019 | Unclear | Unclear | High | High | High |
| Wenbing Chang[60] | 6 | 2019 | Unclear | Unclear | High | High | High |
| Gihun Joo[61] | 1 | 2020 | Low | Unclear | Unclear | High | High |
| Gihun Joo[61] | 2 | 2020 | Low | Unclear | Unclear | High | High |
| Gihun Joo[61] | 3 | 2020 | Low | Unclear | Unclear | High | High |
| Gihun Joo[61] | 4 | 2020 | Low | Unclear | Unclear | High | High |
| Gihun Joo[61] | 5 | 2020 | Low | Unclear | Unclear | High | High |
| Gihun Joo[61] | 6 | 2020 | Low | Unclear | Unclear | High | High |
| Gihun Joo[61] | 7 | 2020 | Low | Unclear | Unclear | High | High |
| Gihun Joo[61] | 8 | 2020 | Low | Unclear | Unclear | High | High |
| V. Sree Hari Rao[62] | 1 | 2020 | Unclear | Unclear | Unclear | High | High |
| V. Sree Hari Rao[62] | 2 | 2020 | Unclear | Unclear | Unclear | High | High |
| Amer M. Johri[63] | 1 | 2021 | Low | Low | Unclear | High | High |
| Amer M. Johri[63] | 2 | 2021 | Low | Low | Unclear | High | High |
| Amer M. Johri[63] | 3 | 2021 | Low | Low | Unclear | High | High |
| Amer M. Johri[63] | 4 | 2021 | Low | Low | Unclear | High | High |
| Amer M. Johri[63] | 5 | 2021 | Low | Low | Unclear | High | High |
| Amer M. Johri[63] | 6 | 2021 | Low | Low | Unclear | High | High |
| Matthew Chun[64] | 1 | 2021 | Low | Unclear | Unclear | High | High |
| Matthew Chun[64] | 2 | 2021 | Low | Unclear | Unclear | High | High |
| Matthew Chun[64] | 3 | 2021 | Low | Unclear | Unclear | High | High |
| Matthew Chun[64] | 4 | 2021 | Low | Unclear | Unclear | High | High |
| Matthew Chun[64] | 5 | 2021 | Low | Unclear | Unclear | High | High |
| Pei-I Zhang[65] | 1 | 2020 | High | Unclear | High | High | High |
| Pei-I Zhang[65] | 2 | 2020 | High | Unclear | High | High | High |
| Pei-I Zhang[65] | 3 | 2020 | High | Unclear | High | High | High |
| Pei-I Zhang[65] | 4 | 2020 | High | Unclear | High | High | High |
| Pei-I Zhang[65] | 5 | 2020 | High | Unclear | High | High | High |
| Pei-I Zhang[65] | 6 | 2020 | High | Unclear | Unclear | High | High |
| Pei-I Zhang[65] | 7 | 2020 | High | Unclear | Unclear | High | High |
| Pei-I Zhang[65] | 8 | 2020 | High | Unclear | Unclear | High | High |
| Pei-I Zhang[65] | 9 | 2020 | High | Unclear | Unclear | High | High |
| Pei-I Zhang[65] | 10 | 2020 | High | Unclear | Unclear | High | High |
| Daniel Lindholm[66] | 1 | 2018 | Low | Low | Unclear | High | High |
| Konstantia Zarkogianni[67] | 1 | 2017 | Unclear | Unclear | Unclear | High | High |
| Konstantia Zarkogianni[67] | 2 | 2017 | Unclear | Unclear | Unclear | High | High |
| Konstantia Zarkogianni[67] | 3 | 2017 | Unclear | Unclear | Unclear | High | High |
| Konstantia Zarkogianni[67] | 4 | 2017 | Unclear | Unclear | Unclear | High | High |
| Konstantia Zarkogianni[67] | 5 | 2017 | Unclear | Unclear | Unclear | High | High |
| Konstantia Zarkogianni[67] | 6 | 2017 | Unclear | Unclear | Unclear | High | High |
| Konstantia Zarkogianni[67] | 7 | 2017 | Unclear | Unclear | Unclear | High | High |
| Konstantia Zarkogianni[67] | 8 | 2017 | Unclear | Unclear | Unclear | High | High |
| Alexandra K. Lee[68] | 1 | 2019 | High | Low | Unclear | High | High |
| Ghalib A. Bello[69] | 1 | 2015 | Low | Unclear | Unclear | High | High |
| Ghalib A. Bello[69] | 2 | 2015 | Low | Unclear | Unclear | High | High |
| Ghalib A. Bello[69] | 3 | 2015 | Low | Unclear | Unclear | High | High |
| Ghalib A. Bello[69] | 4 | 2015 | Low | Unclear | Unclear | High | High |
| Ghalib A. Bello[69] | 5 | 2015 | Low | Unclear | Unclear | High | High |
| Ghalib A. Bello[69] | 6 | 2015 | Low | Unclear | Unclear | High | High |
| Anietie U Andy[70] | 1 | 2021 | High | Unclear | Unclear | High | High |
| Kalliopi Dalakleidi[71] | 1 | 2017 | Unclear | Unclear | Unclear | High | High |
| Kalliopi Dalakleidi[71] | 2 | 2017 | Unclear | Unclear | Unclear | High | High |
| Kalliopi Dalakleidi[71] | 3 | 2017 | Unclear | Unclear | Unclear | High | High |
| Kalliopi Dalakleidi[71] | 4 | 2017 | Unclear | Unclear | Unclear | High | High |
| Sang-Yeong Cho[72] | 1 | 2021 | Low | Unclear | Unclear | High | High |
| Sang-Yeong Cho[72] | 2 | 2021 | Low | Unclear | Unclear | High | High |
| Sang-Yeong Cho[72] | 3 | 2021 | Low | Unclear | Unclear | High | High |
| Sang-Yeong Cho[72] | 4 | 2021 | Low | Unclear | Unclear | High | High |
| Sang-Yeong Cho[72] | 5 | 2021 | Low | Unclear | Unclear | High | High |
| Sang-Yeong Cho[72] | 6 | 2021 | Low | Unclear | Unclear | High | High |
| Sang-Yeong Cho[72] | 7 | 2021 | Low | Unclear | Unclear | High | High |
| Sang-Yeong Cho[72] | 8 | 2021 | Low | Unclear | Unclear | High | High |
| Sang-Yeong Cho[72] | 9 | 2021 | Low | Unclear | Unclear | High | High |
| Sang-Yeong Cho[72] | 10 | 2021 | Low | Unclear | Unclear | High | High |
| Sang-Yeong Cho[72] | 11 | 2021 | Low | Unclear | Unclear | High | High |
| Sang-Yeong Cho[72] | 12 | 2021 | Low | Unclear | Unclear | High | High |
| Sang-Yeong Cho[72] | 13 | 2021 | Low | Unclear | Unclear | High | High |
| Sang-Yeong Cho[72] | 14 | 2021 | Low | Unclear | Unclear | High | High |
| Sang-Yeong Cho[72] | 15 | 2021 | Low | Unclear | Unclear | High | High |
| Sang-Yeong Cho[72] | 16 | 2021 | Low | Unclear | Unclear | High | High |
| Sang-Yeong Cho[72] | 17 | 2021 | Low | Unclear | Unclear | High | High |
| Sang-Yeong Cho[72] | 18 | 2021 | Low | Unclear | Unclear | High | High |
| Sang-Yeong Cho[72] | 19 | 2021 | Low | Unclear | Unclear | High | High |
| Sang-Yeong Cho[72] | 20 | 2021 | Low | Unclear | Unclear | High | High |
| Sang-Yeong Cho[72] | 21 | 2021 | Low | Unclear | Unclear | High | High |
| Sang-Yeong Cho[72] | 22 | 2021 | Low | Unclear | Unclear | High | High |
| Sang-Yeong Cho[72] | 23 | 2021 | Low | Unclear | Unclear | High | High |
| Sang-Yeong Cho[72] | 24 | 2021 | Low | Unclear | Unclear | High | High |
| Andrew Lin[73] | 1 | 2021 | Low | Low | Unclear | High | High |
| Christian Tesche[74] | 1 | 2021 | High | Unclear | Unclear | High | High |
| Christian Tesche[74] | 2 | 2021 | High | Unclear | Unclear | High | High |
| P.Priyanga[75] | 1 | 2020 | High | Unclear | Unclear | High | High |
| P.Priyanga[75] | 2 | 2020 | High | Unclear | Unclear | High | High |
| P.Priyanga[75] | 3 | 2020 | High | Unclear | Unclear | High | High |
| P.Priyanga[75] | 4 | 2020 | High | Unclear | Unclear | High | High |
| P.Priyanga[75] | 5 | 2020 | High | Unclear | Unclear | High | High |
| P.Priyanga[75] | 6 | 2020 | High | Unclear | Unclear | High | High |
| P.Priyanga[75] | 7 | 2020 | High | Unclear | Unclear | High | High |
| Aniruddha Dutta Conceptualization[76] | 1 | 2020 | High | Unclear | High | High | High |
| Aniruddha Dutta Conceptualization[76] | 2 | 2020 | High | Unclear | High | High | High |
| Aniruddha Dutta Conceptualization[76] | 3 | 2020 | High | Unclear | High | High | High |
| Aniruddha Dutta Conceptualization[76] | 4 | 2020 | High | Unclear | High | High | High |
| Aniruddha Dutta Conceptualization[76] | 5 | 2020 | High | Unclear | High | High | High |
| Aniruddha Dutta Conceptualization[76] | 6 | 2020 | High | Unclear | High | High | High |
| Aniruddha Dutta Conceptualization[76] | 7 | 2020 | High | Unclear | High | High | High |
| Aniruddha Dutta Conceptualization[76] | 8 | 2020 | High | Unclear | High | High | High |
| Aniruddha Dutta Conceptualization[76] | 9 | 2020 | High | Unclear | High | High | High |
| Aniruddha Dutta Conceptualization[76] | 10 | 2020 | High | Unclear | High | High | High |
| Aniruddha Dutta Conceptualization[76] | 11 | 2020 | High | Unclear | High | High | High |
| Aniruddha Dutta Conceptualization[76] | 12 | 2020 | High | Unclear | High | High | High |
| Aniruddha Dutta Conceptualization[76] | 13 | 2020 | High | Unclear | High | High | High |
| Aniruddha Dutta Conceptualization[76] | 14 | 2020 | High | Unclear | High | High | High |
| Premanand Tiwari[77] | 1 | 2020 | High | Unclear | Unclear | High | High |
| Premanand Tiwari[77] | 2 | 2020 | High | Unclear | Unclear | High | High |
| Premanand Tiwari[77] | 3 | 2020 | High | Unclear | Unclear | High | High |
| Premanand Tiwari[77] | 4 | 2020 | High | Unclear | Unclear | High | High |
| Premanand Tiwari[77] | 5 | 2020 | High | Unclear | Unclear | High | High |
| Premanand Tiwari[77] | 6 | 2020 | High | Unclear | Unclear | High | High |
| Premanand Tiwari[77] | 7 | 2020 | High | Unclear | Unclear | High | High |
| Yunxing Jiang[78] | 1 | 2021 | Low | Low | Low | High | High |
| Yunxing Jiang[78] | 2 | 2021 | Low | Low | Low | High | High |
| Yunxing Jiang[78] | 3 | 2021 | Low | Low | Low | High | High |
| Yunxing Jiang[78] | 4 | 2021 | Low | Low | Low | High | High |
| Yunxing Jiang[78] | 5 | 2021 | Low | Low | Low | High | High |
| Yunxing Jiang[78] | 6 | 2021 | Low | Low | Low | High | High |
| Yunxing Jiang[78] | 7 | 2021 | Low | Low | Low | High | High |
| Ankush Jamthikar[79] | 1 | 2020 | High | Unclear | Unclear | High | High |
| Che Ngufor[80] | 1 | 2020 | High | Unclear | Unclear | High | High |
| Che Ngufor[80] | 2 | 2020 | High | Unclear | Unclear | High | High |
| Che Ngufor[80] | 3 | 2020 | High | Unclear | High | High | High |
| Che Ngufor[80] | 4 | 2020 | High | Unclear | High | High | High |
| Yaqi Zhang[81] | 1 | 2021 | High | Unclear | Unclear | High | High |
| Yaqi Zhang[81] | 2 | 2021 | High | Unclear | Unclear | High | High |
| Yaqi Zhang[81] | 3 | 2021 | High | Unclear | Unclear | High | High |
| Yaqi Zhang[81] | 4 | 2021 | High | Unclear | Unclear | High | High |
| Yaqi Zhang[81] | 5 | 2021 | High | Unclear | Unclear | High | High |
| Yaqi Zhang[81] | 6 | 2021 | High | Unclear | Unclear | High | High |
| Yaqi Zhang[81] | 7 | 2021 | High | Unclear | Unclear | High | High |
| Yaqi Zhang[81] | 8 | 2021 | Low | Unclear | Unclear | High | High |
| Yaqi Zhang[81] | 9 | 2021 | Low | Unclear | Unclear | High | High |
| Yaqi Zhang[81] | 10 | 2021 | Low | Unclear | Unclear | High | High |
| Yaqi Zhang[81] | 11 | 2021 | Low | Unclear | Unclear | High | High |
| Yaqi Zhang[81] | 12 | 2021 | Low | Unclear | Unclear | High | High |
| Yaqi Zhang[81] | 13 | 2021 | Low | Unclear | Unclear | High | High |
| Yaqi Zhang[81] | 14 | 2021 | Low | Unclear | Unclear | High | High |
| Dongzhe Hong[82] | 1 | 2021 | High | Unclear | Unclear | High | High |
| Dongzhe Hong[82] | 2 | 2021 | High | Unclear | Unclear | High | High |
| Dongzhe Hong[82] | 3 | 2021 | High | Unclear | Unclear | High | High |
| Kinuka Ogata[83] | 1 | 2013 | Low | Low | Unclear | High | High |
| Kinuka Ogata[83] | 2 | 2013 | Low | Low | Unclear | High | High |
| Benjamin A. Goldstein[84] | 1 | 2014 | High | Unclear | Unclear | High | High |
| Benjamin A. Goldstein[84] | 2 | 2014 | High | Unclear | Unclear | High | High |
| Benjamin A. Goldstein[84] | 3 | 2014 | High | Unclear | Unclear | High | High |
| Benjamin A. Goldstein[84] | 4 | 2014 | High | Unclear | Unclear | High | High |
| Benjamin A. Goldstein[84] | 5 | 2014 | High | Unclear | Unclear | High | High |
| Benjamin A. Goldstein[84] | 6 | 2014 | High | Unclear | Unclear | High | High |
| Benjamin A. Goldstein[84] | 7 | 2014 | High | Unclear | Unclear | High | High |
| Benjamin A. Goldstein[84] | 8 | 2014 | High | Unclear | Unclear | High | High |
| Benjamin A. Goldstein[84] | 9 | 2014 | High | Unclear | Unclear | High | High |
| Benjamin A. Goldstein[84] | 10 | 2014 | High | Unclear | Unclear | High | High |
| Benjamin A. Goldstein[84] | 11 | 2014 | High | Unclear | Unclear | High | High |
| Benjamin A. Goldstein[84] | 12 | 2014 | High | Unclear | Unclear | High | High |
| Paolo Emilio Puddu[85] | 1 | 2012 | Low | Low | Unclear | High | High |
| Paolo Emilio Puddu[85] | 2 | 2012 | Low | Low | Unclear | High | High |
| Paolo Emilio Puddu[85] | 3 | 2012 | Low | Low | Unclear | High | High |
| Paolo Emilio Puddu[85] | 4 | 2012 | Low | Low | Unclear | High | High |
| Paolo Emilio Puddu[85] | 5 | 2012 | Low | Low | Unclear | High | High |
| Julian Betancur[86] | 1 | 2018 | Low | Unclear | Unclear | High | High |
| Manish Motwani[87] | 1 | 2016 | Low | Low | Low | High | High |
| Jacob P. VanHouten[88] | 1 | 2014 | High | Unclear | High | High | High |
| Jacob P. VanHouten[88] | 2 | 2014 | High | Unclear | High | High | High |
| Jacob P. VanHouten[88] | 3 | 2014 | High | Unclear | High | High | High |
| Fátima Sánchez-Cabo[89] | 1 | 2020 | Low | Low | Low | High | High |
| Fátima Sánchez-Cabo[89] | 2 | 2020 | Low | Low | Low | High | High |
| Fátima Sánchez-Cabo[89] | 3 | 2020 | Low | Low | Low | High | High |
| Fátima Sánchez-Cabo[89] | 4 | 2020 | Low | Low | Low | High | High |
| Fátima Sánchez-Cabo[89] | 5 | 2020 | Unclear | Unclear | Low | High | High |
| Fátima Sánchez-Cabo[89] | 6 | 2020 | Unclear | Unclear | Low | High | High |
| Fátima Sánchez-Cabo[89] | 7 | 2020 | Unclear | Unclear | Low | High | High |
| Fátima Sánchez-Cabo[89] | 8 | 2020 | Unclear | Unclear | Low | High | High |
| Yafei Wu[90] | 1 | 2020 | Low | Unclear | High | High | High |
| Yafei Wu[90] | 2 | 2020 | Low | Unclear | High | High | High |
| Yafei Wu[90] | 3 | 2020 | Low | Unclear | High | High | High |
| Georgios Christopoulos[91] | 1 | 2020 | Low | Low | Unclear | High | High |
| Georgios Christopoulos[91] | 2 | 2020 | Low | Low | Unclear | High | High |
| Georgios Christopoulos[91] | 3 | 2020 | Low | Low | Unclear | High | High |
| Georgios Christopoulos[91] | 4 | 2020 | Low | Low | Unclear | High | High |
| Georgios Christopoulos[91] | 5 | 2020 | Low | Low | Unclear | High | High |
| Georgios Christopoulos[91] | 6 | 2020 | Low | Low | Unclear | High | High |
| Georgios Christopoulos[91] | 7 | 2020 | Low | Low | Unclear | High | High |
| Georgios Christopoulos[91] | 8 | 2020 | Low | Low | Unclear | High | High |
| Renate M. Hoogeveen[92] | 1 | 2020 | High | Low | Unclear | High | High |
| Renate M. Hoogeveen[92] | 2 | 2020 | High | Low | Unclear | High | High |
| Agni Orfanoudaki[93] | 1 | 2019 | Low | Low | Unclear | High | High |
| Agni Orfanoudaki[93] | 2 | 2019 | Low | Low | Unclear | High | High |
| Agni Orfanoudaki[93] | 3 | 2019 | Low | Low | Unclear | High | High |
| Agni Orfanoudaki[93] | 4 | 2019 | Low | Low | Unclear | High | High |
| Agni Orfanoudaki[93] | 5 | 2019 | Low | Low | Unclear | High | High |
| Agni Orfanoudaki[93] | 6 | 2019 | Low | Low | Unclear | High | High |
| Agni Orfanoudaki[93] | 7 | 2019 | Low | Low | Unclear | High | High |
| Agni Orfanoudaki[93] | 8 | 2019 | Low | Low | Unclear | High | High |
| Agni Orfanoudaki[93] | 9 | 2019 | Low | Low | Unclear | High | High |
| Agni Orfanoudaki[93] | 10 | 2019 | Low | Low | Unclear | High | High |
| Agni Orfanoudaki[93] | 11 | 2019 | Low | Low | Unclear | High | High |
| Agni Orfanoudaki[93] | 12 | 2019 | Low | Low | Unclear | High | High |
| Laila R Bekhet[94] | 1 | 2018 | High | Unclear | Unclear | High | High |
| Laila R Bekhet[94] | 2 | 2018 | High | Unclear | Unclear | High | High |
| Christoph Nowak[95] | 1 | 2018 | Low | Low | Unclear | High | High |
| Christoph Nowak[95] | 2 | 2018 | Low | Low | Unclear | High | High |
| Alexandros C. Dimopoulos[96] | 1 | 2018 | Low | Unclear | Unclear | High | High |
| Alexandros C. Dimopoulos[96] | 2 | 2018 | Low | Unclear | Unclear | High | High |
| Alexandros C. Dimopoulos[96] | 3 | 2018 | Low | Unclear | Unclear | High | High |
| Juan Zhao[97] | 1 | 2019 | High | Unclear | Unclear | High | High |
| Juan Zhao[97] | 2 | 2019 | High | Unclear | Unclear | High | High |
| Juan Zhao[97] | 3 | 2019 | High | Unclear | Unclear | High | High |
| Juan Zhao[97] | 4 | 2019 | High | Unclear | Unclear | High | High |
| Juan Zhao[97] | 5 | 2019 | High | Unclear | Unclear | High | High |
| Juan Zhao[97] | 6 | 2019 | High | Unclear | Unclear | High | High |
| Shinya Suzuki[98] | 1 | 2019 | Low | Low | Unclear | High | High |
| Shinya Suzuki[98] | 2 | 2019 | Low | Low | Unclear | High | High |
| Shinya Suzuki[98] | 3 | 2019 | Low | Low | Unclear | High | High |
| Shinya Suzuki[98] | 4 | 2019 | Low | Low | Unclear | High | High |
| Shinya Suzuki[98] | 5 | 2019 | Low | Low | High | High | High |
| Shinya Suzuki[98] | 6 | 2019 | Low | Low | High | High | High |
| Shinya Suzuki[98] | 7 | 2019 | Low | Low | Unclear | High | High |
| Shinya Suzuki[98] | 8 | 2019 | Low | Low | Unclear | High | High |
| Shinya Suzuki[98] | 9 | 2019 | Low | Low | Unclear | High | High |
| Shinya Suzuki[98] | 10 | 2019 | Low | Low | Unclear | High | High |
| Shinya Suzuki[98] | 11 | 2019 | Low | Low | Unclear | High | High |
| Shinya Suzuki[98] | 12 | 2019 | Low | Low | Unclear | High | High |
| Sabrina Mezzatesta[99] | 1 | 2019 | Unclear | Unclear | Unclear | High | High |
| Sabrina Mezzatesta[99] | 2 | 2019 | Unclear | Unclear | Unclear | High | High |
| Sabrina Mezzatesta[99] | 3 | 2019 | Unclear | Unclear | Unclear | High | High |
| Sabrina Mezzatesta[99] | 4 | 2019 | Unclear | Unclear | Unclear | High | High |
| Sabrina Mezzatesta[99] | 5 | 2019 | Unclear | Unclear | Unclear | High | High |
| Sabrina Mezzatesta[99] | 6 | 2019 | Unclear | Unclear | Unclear | High | High |
| Sabrina Mezzatesta[99] | 7 | 2019 | Unclear | Unclear | Unclear | High | High |
| Sabrina Mezzatesta[99] | 8 | 2019 | Unclear | Unclear | Unclear | High | High |
| Sabrina Mezzatesta[99] | 9 | 2019 | Unclear | Unclear | Unclear | High | High |
| Sabrina Mezzatesta[99] | 10 | 2019 | Unclear | Unclear | Unclear | High | High |
| Sabrina Mezzatesta[99] | 11 | 2019 | Unclear | Unclear | Unclear | High | High |
| Sabrina Mezzatesta[99] | 12 | 2019 | Unclear | Unclear | Unclear | High | High |
| Sabrina Mezzatesta[99] | 13 | 2019 | Unclear | Unclear | Unclear | High | High |
| Sabrina Mezzatesta[99] | 14 | 2019 | Unclear | Unclear | Unclear | High | High |
| Sabrina Mezzatesta[99] | 15 | 2019 | Unclear | Unclear | Unclear | High | High |
| Sabrina Mezzatesta[99] | 16 | 2019 | Unclear | Unclear | Unclear | High | High |
| Sabrina Mezzatesta[99] | 17 | 2019 | Unclear | Unclear | Unclear | High | High |
| Sabrina Mezzatesta[99] | 18 | 2019 | Unclear | Unclear | Unclear | High | High |
| Sabrina Mezzatesta[99] | 19 | 2019 | Unclear | Unclear | Unclear | High | High |
| Sabrina Mezzatesta[99] | 20 | 2019 | Unclear | Unclear | Unclear | High | High |
| Sabrina Mezzatesta[99] | 21 | 2019 | Unclear | Unclear | Unclear | High | High |
| Sabrina Mezzatesta[99] | 22 | 2019 | Unclear | Unclear | Unclear | High | High |
| Sabrina Mezzatesta[99] | 23 | 2019 | Unclear | Unclear | Unclear | High | High |
| Sabrina Mezzatesta[99] | 24 | 2019 | Unclear | Unclear | Unclear | High | High |
| Sabrina Mezzatesta[99] | 25 | 2019 | Unclear | Unclear | Unclear | High | High |
| Sabrina Mezzatesta[99] | 26 | 2019 | Unclear | Unclear | Unclear | High | High |
| Sabrina Mezzatesta[99] | 27 | 2019 | Unclear | Unclear | Unclear | High | High |
| Sabrina Mezzatesta[99] | 28 | 2019 | Unclear | Unclear | Unclear | High | High |
| Sabrina Mezzatesta[99] | 29 | 2019 | Unclear | Unclear | Unclear | High | High |
| Sabrina Mezzatesta[99] | 30 | 2019 | Unclear | Unclear | Unclear | High | High |
| Sabrina Mezzatesta[99] | 31 | 2019 | Unclear | Unclear | Unclear | High | High |
| Sabrina Mezzatesta[99] | 32 | 2019 | Unclear | Unclear | Unclear | High | High |
| Sabrina Mezzatesta[99] | 33 | 2019 | Unclear | Unclear | Unclear | High | High |
| Sabrina Mezzatesta[99] | 34 | 2019 | Unclear | Unclear | Unclear | High | High |
| Sabrina Mezzatesta[99] | 35 | 2019 | Unclear | Unclear | Unclear | High | High |
| Sabrina Mezzatesta[99] | 36 | 2019 | Unclear | Unclear | Unclear | High | High |
| Sabrina Mezzatesta[99] | 37 | 2019 | Unclear | Unclear | Unclear | High | High |
| Sabrina Mezzatesta[99] | 38 | 2019 | Unclear | Unclear | Unclear | High | High |
| Sabrina Mezzatesta[99] | 39 | 2019 | Unclear | Unclear | Unclear | High | High |
| Sabrina Mezzatesta[99] | 40 | 2019 | Unclear | Unclear | Unclear | High | High |
| Sabrina Mezzatesta[99] | 41 | 2019 | Unclear | Unclear | Unclear | High | High |
| Sabrina Mezzatesta[99] | 42 | 2019 | Unclear | Unclear | Unclear | High | High |
| Sabrina Mezzatesta[99] | 43 | 2019 | Unclear | Unclear | Unclear | High | High |
| Sabrina Mezzatesta[99] | 44 | 2019 | Unclear | Unclear | Unclear | High | High |
| Sabrina Mezzatesta[99] | 45 | 2019 | Unclear | Unclear | Unclear | High | High |
| Sabrina Mezzatesta[99] | 46 | 2019 | Unclear | Unclear | Unclear | High | High |
| Sabrina Mezzatesta[99] | 47 | 2019 | Unclear | Unclear | Unclear | High | High |
| Sabrina Mezzatesta[99] | 48 | 2019 | Unclear | Unclear | Unclear | High | High |
| Sabrina Mezzatesta[99] | 49 | 2019 | Unclear | Unclear | Unclear | High | High |
| Sabrina Mezzatesta[99] | 50 | 2019 | Unclear | Unclear | Unclear | High | High |
| Sabrina Mezzatesta[99] | 51 | 2019 | Unclear | Unclear | Unclear | High | High |
| Sabrina Mezzatesta[99] | 52 | 2019 | Unclear | Unclear | Unclear | High | High |
| Sabrina Mezzatesta[99] | 53 | 2019 | Unclear | Unclear | Unclear | High | High |
| Sabrina Mezzatesta[99] | 54 | 2019 | Unclear | Unclear | Unclear | High | High |
| Sabrina Mezzatesta[99] | 55 | 2019 | Unclear | Unclear | Unclear | High | High |
| Sabrina Mezzatesta[99] | 56 | 2019 | Unclear | Unclear | Unclear | High | High |
| Sabrina Mezzatesta[99] | 57 | 2019 | Unclear | Unclear | Unclear | High | High |
| Sabrina Mezzatesta[99] | 58 | 2019 | Unclear | Unclear | Unclear | High | High |
| Sabrina Mezzatesta[99] | 59 | 2019 | Unclear | Unclear | Unclear | High | High |
| Sabrina Mezzatesta[99] | 60 | 2019 | Unclear | Unclear | Unclear | High | High |
| Sabrina Mezzatesta[99] | 61 | 2019 | Unclear | Unclear | Unclear | High | High |
| Sabrina Mezzatesta[99] | 62 | 2019 | Unclear | Unclear | Unclear | High | High |
| Sabrina Mezzatesta[99] | 63 | 2019 | Unclear | Unclear | Unclear | High | High |
| Sabrina Mezzatesta[99] | 64 | 2019 | Unclear | Unclear | Unclear | High | High |
| Sabrina Mezzatesta[99] | 65 | 2019 | Unclear | Unclear | Unclear | High | High |
| Sabrina Mezzatesta[99] | 66 | 2019 | Unclear | Unclear | Unclear | High | High |
| Sabrina Mezzatesta[99] | 67 | 2019 | Unclear | Unclear | Unclear | High | High |
| Sabrina Mezzatesta[99] | 68 | 2019 | Unclear | Unclear | Unclear | High | High |
| Sabrina Mezzatesta[99] | 69 | 2019 | Unclear | Unclear | Unclear | High | High |
| Sabrina Mezzatesta[99] | 70 | 2019 | Unclear | Unclear | Unclear | High | High |
| Ji Min Sung[100] | 1 | 2019 | Low | Low | Unclear | High | High |
| Ji Min Sung[100] | 2 | 2019 | Low | Low | Unclear | High | High |
| Jose A. Quesada[101] | 1 | 2019 | High | Unclear | Unclear | High | High |
| Jose A. Quesada[101] | 2 | 2019 | High | Unclear | Unclear | High | High |
| Jose A. Quesada[101] | 3 | 2019 | High | Unclear | Unclear | High | High |
| Jose A. Quesada[101] | 4 | 2019 | High | Unclear | Unclear | High | High |
| Jose A. Quesada[101] | 5 | 2019 | High | Unclear | Unclear | High | High |
| Jose A. Quesada[101] | 6 | 2019 | High | Unclear | Unclear | High | High |
| Jose A. Quesada[101] | 7 | 2019 | High | Unclear | Unclear | High | High |
| Jose A. Quesada[101] | 8 | 2019 | High | Unclear | Unclear | High | High |
| Jose A. Quesada[101] | 9 | 2019 | High | Unclear | Unclear | High | High |
| Jose A. Quesada[101] | 10 | 2019 | High | Unclear | Unclear | High | High |
| Jose A. Quesada[101] | 11 | 2019 | High | Unclear | Unclear | High | High |
| Jose A. Quesada[101] | 12 | 2019 | High | Unclear | Unclear | High | High |
| Jose A. Quesada[101] | 13 | 2019 | High | Unclear | Unclear | High | High |
| Jose A. Quesada[101] | 14 | 2019 | High | Unclear | Unclear | High | High |
| Randall W. Grout[102] | 1 | 2021 | High | Unclear | Unclear | High | High |
| Randall W. Grout[102] | 2 | 2021 | High | Unclear | Unclear | High | High |
| Shelda Sajeev[103] | 1 | 2021 | Low | Unclear | Unclear | High | High |
| Shelda Sajeev[103] | 2 | 2021 | Low | Unclear | Unclear | High | High |
| Shelda Sajeev[103] | 3 | 2021 | Low | Unclear | Unclear | High | High |
| Shelda Sajeev[103] | 4 | 2021 | Low | Unclear | Unclear | High | High |
| Shelda Sajeev[103] | 5 | 2021 | Low | Unclear | Unclear | High | High |
| Shelda Sajeev[103] | 6 | 2021 | Low | Unclear | Unclear | High | High |
| Shelda Sajeev[103] | 7 | 2021 | Low | Unclear | Unclear | High | High |
| Shelda Sajeev[103] | 8 | 2021 | Low | Unclear | Unclear | High | High |
| David de Gonzalo-Calvo[104] | 1 | 2020 | High | Low | Unclear | High | High |
| In-Soo Kim[105] | 1 | 2020 | High | Unclear | Unclear | High | High |
| In-Soo Kim[105] | 2 | 2020 | High | Unclear | Unclear | High | High |
| In-Soo Kim[105] | 3 | 2020 | High | Unclear | Unclear | High | High |
| In-Soo Kim[105] | 4 | 2020 | High | Unclear | Unclear | High | High |
| In-Soo Kim[105] | 5 | 2020 | High | Unclear | Unclear | High | High |
| In-Soo Kim[105] | 6 | 2020 | High | Unclear | Unclear | High | High |
| Michael SCHREMPF[106] | 1 | 2021 | High | Unclear | Unclear | High | High |
| Michael SCHREMPF[106] | 2 | 2021 | High | Unclear | Unclear | High | High |
| Michael SCHREMPF[106] | 3 | 2021 | High | Unclear | Unclear | High | High |
| Michael SCHREMPF[106] | 4 | 2021 | High | Unclear | Unclear | High | High |
| Simon Nusinovici[107] | 1 | 2020 | Low | Unclear | Unclear | High | High |
| Simon Nusinovici[107] | 2 | 2020 | Low | Unclear | Unclear | High | High |
| Simon Nusinovici[107] | 3 | 2020 | Low | Unclear | Unclear | High | High |
| Simon Nusinovici[107] | 4 | 2020 | Low | Unclear | Unclear | High | High |
| Simon Nusinovici[107] | 5 | 2020 | Low | Unclear | Unclear | High | High |
| Simon Nusinovici[107] | 6 | 2020 | Low | Unclear | Unclear | High | High |
| Luca Navarini[108] | 1 | 2020 | Low | Unclear | Unclear | High | High |
| Luca Navarini[108] | 2 | 2020 | Low | Unclear | Unclear | High | High |
| Luca Navarini[108] | 3 | 2020 | Low | Unclear | Unclear | High | High |
| Luca Navarini[108] | 4 | 2020 | Low | Unclear | Unclear | High | High |
| Luca Navarini[108] | 5 | 2020 | Low | Unclear | Unclear | High | High |
| Luca Navarini[108] | 6 | 2020 | Low | Unclear | Unclear | High | High |
| Divneet Mandair[109] | 1 | 2020 | High | Unclear | Unclear | High | High |
| Divneet Mandair[109] | 2 | 2020 | High | Unclear | Unclear | High | High |
| Divneet Mandair[109] | 3 | 2020 | High | Unclear | Unclear | High | High |
| Divneet Mandair[109] | 4 | 2020 | High | Unclear | Unclear | High | High |
| Divneet Mandair[109] | 5 | 2020 | High | Unclear | Unclear | High | High |
| Divneet Mandair[109] | 6 | 2020 | High | Unclear | Unclear | High | High |
| Divneet Mandair[109] | 7 | 2020 | High | Unclear | Unclear | High | High |
| Divneet Mandair[109] | 8 | 2020 | High | Unclear | Unclear | High | High |

**Text 2 Search strategies of AI/ML assessment guidelines or tools**

Pubmed

Assessment tool [Text Word] OR Assessment score [Text Word] OR Research guideline [Text Word] OR Criteria [Text Word] OR Scale [Text Word]

AND

Machine learning [MeSH Terms] OR Artificial intelligence [MeSH Terms] OR Machine learning [Text Word] OR Artificial intelligence [Text Word]

AND

Prediction [Text Word] OR Validation [Text Word] OR Risk [MeSH Terms] OR Bias [MeSH Terms] OR Quality[Title/Abstract]

AND

"1985/01/01"[Date - Publication]: "2023/10/31"[Date - Publication]

**Fig. S1. The flow diagram for literature search in the assessment guidelines or tools in the field of medical AI/ML research**


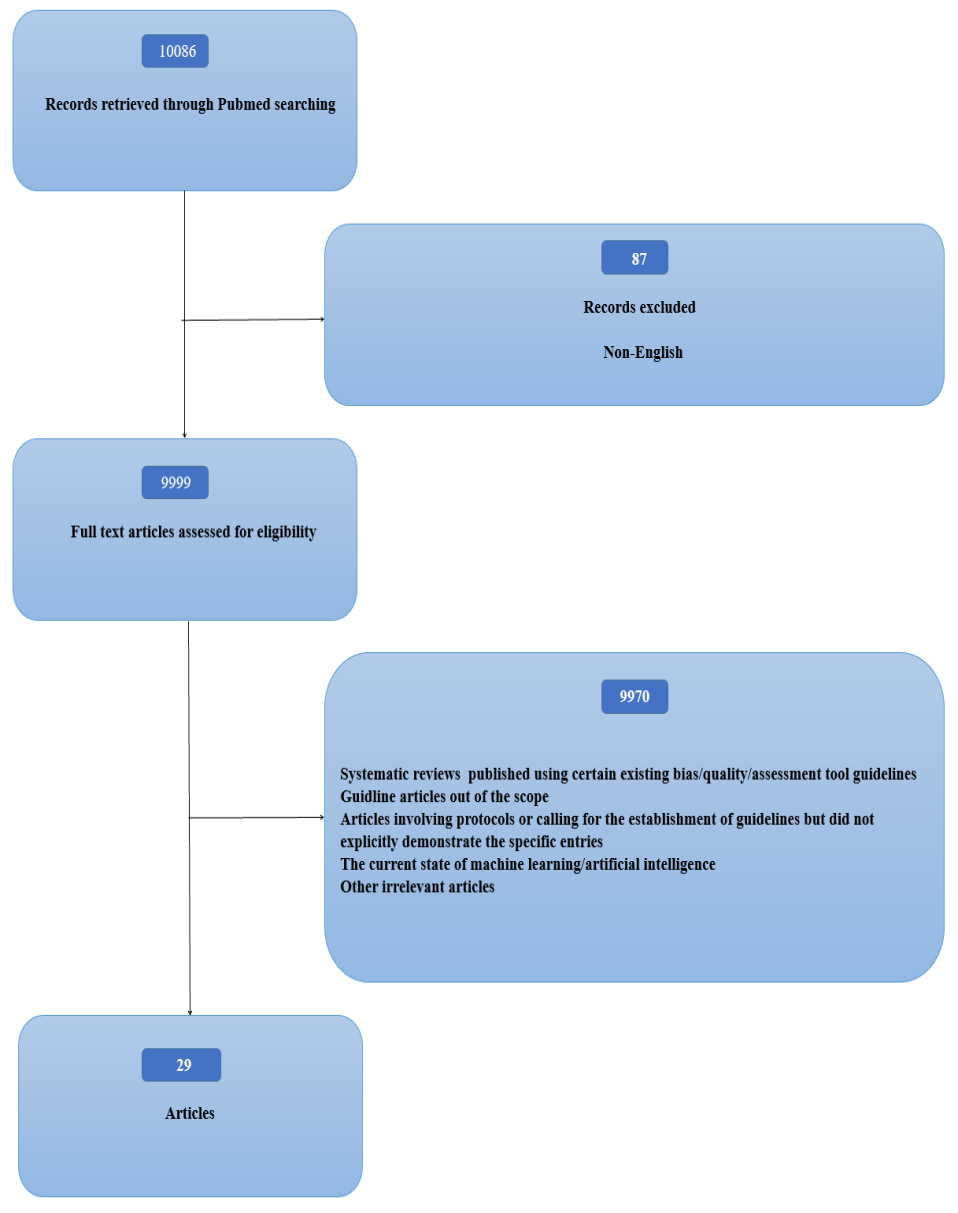


**Table S6. The characteristics of assessment guidelines or tools in the field of medical AI/ML research**

|  | **Year** | **Abbreviation** | **Target Type** | **Target Domain** | **Evaluation Method** | **Evaluation Type** | **Evaluation Content** | **Items Number** | **Generation Method** |
| --- | --- | --- | --- | --- | --- | --- | --- | --- | --- |
| [110] | 2023 | - | Radiology AI software | Universal | Checklist/  framework | Developing Quality | Company Information Implementation and Application Required Resource AI Ethics Study Design Model Performance Data Acquisition and Processing Information of Cost | 37 items | Uncertain |
| [111] | 2023 | DQM | Diagnostic models | Universal | Checklist/  framework | Developing Quality | Clinical Implication Implementation and Application Study Design Data Acquisition and Processing AI Ethics Organization Factors Model Performance | 43 items | Roundtable discussion by experts +combination with the existing standards |
| [112] | 2023 | MAIC-10 | Medical images models | Universal | score(0-10) | Reporting Quality | Project Rationale/Clinical implication Study Design AI Ethics Data Acquisition and Processing  Model Performance Implementation and Application  Replicability | 10 items | literature review of existing guidelines + votation by a panel of experts |
| [113] | 2023 | BS30440 | The use of AI in healthcare | Universal | Checklist/  framework | Developing Quality | Project Rationale/Clinical implication Study Design Human Factors Carbon Impact AI Ethics Required Resource and Cost  Model Performance Monitoring | 18 items | literature review of existing guideline + consultation by a panel of experts  +participation of patients and public representatives |
| [114] | 2023 | AI-TMM | Trustworth | Universal | Checklist/  framework | Developing Quality | Data Acquisition and Processing AI Ethics Accountability Replicability Model Performance Sociatal well being | 65 items | Uncertain |
| [115] | 2023 | APPRAISE-AI | Clinical Decision Support | Universal | Score  (0-100) | Developing Quality Reporting Quality | Project Rationale/Clinical implication  Study Design Statistical Methods Data Acquisition and Processing Model Performance Implementation and Application AI Ethics Replicability | 24 items | literature review of existing guideline + discussion by a panel of experts |
| [116] | 2023 | Consolidated Guidelines | Prognostic and Diagnostic Models | Universal | Checklist/  framework | Reporting Quality | Project Rationale/Clinical implication Study Design Statistical Methods Data Acquisition and Processing Model Performance Implementation and Application AI Ethics Replicability | 37 items | Literature review of existing guidelines |
| [117] | 2023 | CLEAR | Radiomics research | Universal | Checklist/  framework | Reporting Quality | Project Rationale/Clinical implication Study Design  Statistical Methods Data Acquisition and Processing Model Performance Implementation and Application Replicability | 58 items | Literature review of existing guideline + International expert consensus |
| [118] | 2022 | - | Prediction Models | Cardiovascular disease | Checklist/  framework | Developing Quality Reporting Quality | Project Rationale/Clinical implication Implementation and Application Study Design Data Acquisition and Processing AI Ethics Replicability Model Performance | 12 items | Uncertain |
| [119] | 2022 | CLEAR Derm | Medical images models | Dermatology | Checklist/  framework | Reporting Quality | Clinical implication Study Design Data Acquisition and Processing Risk of Bias Model Performance Implementation and Application Replicability | 25 items | literature review of existing guideline + discussion by a panel of experts |
| [120] | 2022 | DECIDE-AI | Decision support systems | Universal | Checklist/  framework | Reporting Quality | Project Rationale/Clinical implication Study Design Implementation and Application Statistical Methods Data Acquisition and Processing Model Performance AI Ethics Risk of Bias | 38 items | International expert consensus |
| [121] | 2022 | RELAINCE Guidelines | AI models | Nuclear Medicine | Checklist/  framework | Developing Quality Reporting Quality | Project Rationale/Clinical implication Study Design Implementation and Application Statistical Methods Data Acquisition and Processing Model Performance AI Ethics Risk of Bias Replicability Monitoring | 19 items | Uncertain |
| [11] | 2021 | - | Prediction Models | Cardiovascular disease | Checklist/  framework | Developing Quality Reporting Quality | Project Rationale/Clinical implication Implementation and Application Model Performance Study Design AI Ethics Replicability Risk of Bias Cost Effectiveness | 22 items | Development from existing guidelines |
| [122] | 2021 | DOME | Supervised ML models | Biology | Checklist/  framework | Developing Quality Reporting Quality | Study Design Statistical Methods Data Acquisition and Processing Model Performance AI Ethics | 37 items | International expert consensus |
| [123] | 2021 | CAIR | Clinical AI Research | Universal | Checklist/  framework | Reporting Quality | Project Rationale/Clinical implication Statistical Methods Model Performance Data Acquisition and Processing Model Performance AI Ethics Replicability | Uncertain | Author summary |
| [124] | 2021 | AIMe | AI research | Biomedical field | Checklist/  framework | Reporting Quality | Project Rationale/Clinical implication Implementation and Application Statistical Methods Data Acquisition and Processing Model Performance Risk of Bias Replicability | Uncertain | Design from a panel of experts  + feedback by researchers |
| [125] | 2021 | - | AI research | Dental field | Checklist/  framework | Developing Quality Reporting Quality | Project Rationale/Clinical implication Study Design Implementation and Application Statistical Methods Data Acquisition and Processing Required Resources Model Performance Risk of Bias Replicability | 30 items | Expert consensus |
| [126] | 2021 | - | Clinical application | Universal | Checklist/  framework | Developing Quality | Project Rationale/Clinical implication Implementation and Application Study Design Statistical Methods Data Acquisition and Processing Model Performance AI Ethics Risk of Bias Replicability | 12 items | Literature review of existing guidelines |
| [127] | 2020 | MI-CLAIM | AI models | Universal | Checklist/  framework | Reporting Quality | Project Rationale/Clinical implication Study Design  Data Acquisition and Processing Model Performance AI Ethics Replicability | 22 items | Uncertain |
| [128] | 2020 | CONSORT-AI Extension | Clinical trial reports | Universal | Checklist/  framework | Reporting Quality | Project Rationale/Clinical implication Study Design Statistical Methods Data Acquisition and Processing Implementation and Application AI Ethics Intervention | 14 items | literature review of existing guidelines + votation by a panel of experts |
| [129] | 2020 | AI-TREE | ML/AI health research | Universal | Checklist/  framework | Developing Quality Reporting Quality | Project Rationale/Clinical implication Study Design Statistical Methods Required Resources Data Acquisition and Processing Implementation and Application AI Ethics Replicability | 20 items | Discussion by experts |
| [130] | 2020 | PRIME | Medical images models | Cardiovascular field | Checklist/  framework | Reporting Quality | Project Rationale/Clinical implication Study Design Implementation and Application Statistical Methods Data Acquisition and Processing Model Performance AI Ethics Replicability | 28 items | Uncertain |
| [131] | 2020 | SPIRIT-AI Extension | Clinical AI intervention trials | Universal | Checklist/  framework | Reporting Quality | Project Rationale/Clinical implication Study Design Statistical Methods Data Acquisition and Processing Implementation and Application Intervention | 15 items | Literature review of existing guidelines +steering group and international expert consensus |
| [132] | 2020 | - | AI Research | Universal | Checklist/  framework | Reporting Quality | Study Design Data Acquisition and Processing Model Performance Replicability Implementation and Application | 5 items | Uncertain |
| [133] | 2020 | - | Clinical AI Research | Universal | Checklist/  framework | Reporting Quality | Project Rationale/Clinical implication  Study Design  Statistical Methods  Data Acquisition and Processing Model Performance  Implementation and Application Risk of Bias  Replicability | uncertain | Development from existing guidelines |
| [30] | 2019 | PROBAST | Prediction Model | Universal | Checklist/  framework | Developing Quality Reporting Quality | Study Design Statistical Methods Data Acquisition and Processing Model Performance Risk of Bias | 20 items | International expert consensus |
| [134] | 2017 | RQS | Radiomics research | Universal | Checklist/  framework | Developing Quality Reporting Quality | Implementation and Application Statistical Methods Data Acquisition and Processing Model Performance Cost-effectiveness Replicability | 16 items | Uncertain |
| [135] | 2016 | - | Prediction Models | Biomedical Research | Checklist/  framework | Developing Quality Reporting Quality | Project Rationale/Clinical implication Study Design Statistical Methods Data Acquisition and Processing Model Performance | 18 items | Recommendation by multidisciplinary experts panel |
| [136] | 2015 | TRIPOD statement | Prognosis or diagnosis models | Universal | Checklist/  framework | Reporting Quality | Project Rationale/Clinical implication Study Design Statistical Methods Data Acquisition and Processing Model Performance Implementation and Application | 37 items | International expert consensus |

**Table S7. The characteristics of 10 recommended models**

| **Author; year** | **Independent Validation Score (IVS)** | | | | | **Algorithm** | **Performance of models** | **Variables** | **Outcomes** | **Datasets** |
| --- | --- | --- | --- | --- | --- | --- | --- | --- | --- | --- |
|  | **1** | **2** | **3** | **4** | **5** |  |  |  |  |  |
| Daniel Lindholm, 2018 | Ⅱ | Ⅱ | Ⅱ | Ⅱ | Ⅱ | COX(GBM) | AUC: 0.85;  Calibartion plot: good | age, sex, body mass index, alcohol consumption, smoking status, self-reported myocardial infarction, diabetes mellitus, prevalent chronic renal failure, prevalent coronary heart disease, leg bioimpedance, systolic blood pressure, diastolic blood pressure, antihypertensive treatment | Heart failure | UK Biobank |
| Sang-Yeong Cho, 2021 | Ⅱ | Ⅱ | Ⅱ | Ⅱ | Ⅱ | LogR | AUC: 0.749;  Calibartion plot: good | age, sex, smoking status, history of diabetes, systolic blood pressure, total cholesterol, high-density lipoprotein cholesterol, antihypertensive medication use | Hard ASCVD | PCE |
|  | Ⅱ | Ⅱ | Ⅱ | Ⅱ | Ⅱ | NN | AUC: 0.748;  Calibartion plot: good |  |  |  |
| Yunxing Jiang, 2021 | Ⅱ | Ⅱ | Ⅱ | Ⅱ | Ⅱ | DT | AUC: 0.770;  Calibartion plot: good | age, sex, waistline, body mass index, family history of hypertension, family history of diabetes, current smoker, alcohol drinking, dyslipidemia, metabolic syndrome, systolic blood pressure, fasting plasma glucose, triglyceride, total cholesterol, high-density lipoprotein, body adiposity index, LHR(LDL/HDL ratio), nonesterified fatty acid, hs-CRP, adiponectin, interleukin 6, insulin | CVD | Kazakh population of China |
|  | Ⅱ | Ⅱ | Ⅱ | Ⅱ | Ⅱ | RF | AUC: 0.840;  Calibartion plot: good |  |  |  |
|  | Ⅱ | Ⅱ | Ⅱ | Ⅱ | Ⅱ | KNN | AUC: 0.845;  Calibartion plot: good |  |  |  |
|  | Ⅱ | Ⅱ | Ⅱ | Ⅱ | Ⅱ | GNB | AUC: 0.791;  Calibartion plot: good |  |  |  |
|  | Ⅱ | Ⅱ | Ⅱ | Ⅱ | Ⅱ | SVM | AUC: 0.868;  Calibartion plot: good |  |  |  |
|  | Ⅱ | Ⅱ | Ⅱ | Ⅱ | Ⅱ | XGBoost | AUC: 0.804;  Calibartion plot: good |  |  |  |
|  | Ⅱ | Ⅱ | Ⅱ | Ⅱ | Ⅱ | LogR | AUC: 0.872;  Calibartion plot: good |  |  |  |
